# Molecular determinants of multidrug-resistant tuberculosis in Sierra Leone

**DOI:** 10.1128/spectrum.02405-23

**Published:** 2024-01-30

**Authors:** Harriet N. A. Blankson, Rashidatu Fouad Kamara, Ivan Barilar, Sönke Andres, Ousman S. Conteh, Tobias Dallenga, Lynda Foray, Florian Maurer, Katharina Kranzer, Christian Utpatel, Stefan Niemann

**Affiliations:** 1 Molecular and Experimental Mycobacteriology, Research Center Borstel Leibniz Lung Center, Borstel, Germany; 2 German Center for Infection Research, Partner Site Hamburg-Lübeck-Borstel-Reims, Borstel, Germany; 3 School of Biomedical and Allied Health Sciences, College of Health Sciences, University of Ghana, Korle-Bu, Accra, Ghana; 4 National Leprosy and Tuberculosis Control Programme Sierra Leone, Freetown, Sierra Leone; 5 National and WHO Supranational Reference Center for Mycobacteria, Research Center Borstel Leibniz Lung Center, Borstel, Germany; 6 Cellular Microbiology, Research Center Borstel Leibniz Lung Center, Borstel, Germany; 7 Institute of Medical Microbiology, Virology and Hygiene, University Medical Center Hamburg-Eppendorf, Hamburg, Germany; 8 Clinical Research Department, London School of Hygiene and Tropical Medicine, London, United Kingdom; University of Manitoba, Winnipeg, Manitoba, Canada

**Keywords:** multidrug resistance, *Mycobacterium tuberculosis*, Sierra Leone, tuberculosis

## Abstract

Multidrug-resistant tuberculosis (MDR-TB) management has become a serious global health challenge. Understanding its epidemic determinants on the regional level is crucial for developing effective control measures. We used whole genome sequencing data of 238 of *Mycobacterium tuberculosis* complex (MTBC) strains to determine drug resistance profiles, phylogeny, and transmission dynamics of MDR/rifampicin-resistant (RR) MTBC strains from Sierra Leone. Forty-two strains were classified as RR, 196 as MDR, 5 were resistant to bedaquiline (BDQ) and clofazimine (CFZ), but none was found to be resistant to fluoroquinolones. Sixty-one (26%) strains were resistant to all first-line drugs, three of which had additional resistance to BDQ/CFZ. The strains were classified into six major MTBC lineages (L), with strains of L4 being the most prevalent, 62% (*n* = 147), followed by L6 (*Mycobacterium africanum*) strains, (21%, *n* = 50). The overall clustering rate (using ≤d12 single-nucleotide polymorphism threshold) was 44%, stratified into 31 clusters ranging from 2 to 16 strains. The largest cluster (*n* = 16) was formed by sublineage 2.2.1 Beijing Ancestral 3 strains, which developed MDR several times. Meanwhile, 10 of the L6 strains had a primary MDR transmission. We observed a high diversity of drug resistance mutations, including borderline resistance mutations to isoniazid and rifampicin, and mutations were not detected by commercial assays. In conclusion, one in five strains investigated was resistant to all first-line drugs, three of which had evidence of BDQ/CFZ resistance. Implementation of interventions such as rapid diagnostics that prevent further resistance development and stop MDR-TB transmission chains in the country is urgently needed.

**IMPORTANCE:**

A substantial proportion of MDR-TB strains in Sierra Leone were resistant against all first line drugs; however this makes the all-oral-six-month BPaLM regimen or other 6-9 months all oral regimens still viable, mainly because there was no FQ resistance.Resistance to BDQ was detected, as well as RR, due to mutations outside of the hotspot region. While the prevalence of those resistances was low, it is still cause for concern and needs to be closely monitored.

## INTRODUCTION

Tuberculosis (TB) is among the leading causes of death from a single infectious agent, accounting for 1.6 million deaths in 2021 (1). TB is caused by pathogens of the *Mycobacterium tuberculosis* complex (MTBC), consisting of *Mycobacterium tuberculosis sensu stricto* (*Mtb*), *Mycobacterium africanum* (*Maf*) (2), *Mycobacterium bovis*, and strains of other animal-adapted species (3–5). *Mtb* strains cause most human diseases globally, while *Maf strains* are responsible for 20%–40% of diseases in West Africa (Benin—37%, Ghana—20%, Ginneau Bissau—47%, and Sierra Leone—23%) (6–11). MTBC strains can be further classified into nine main lineages (L) and several sublineages using single-nucleotide polymorphism (SNP)-based barcoding classification (12). *Mtb* strains belong to L1–L4, L7, and L8, while *Maf* belongs to L5, L6, and L9 (11).

Drug-susceptible TB is treatable with 6 months of a standard drug regimen composed of isoniazid (INH), rifampicin (RIF), pyrazinamide (PZA), and ethambutol (EMB) (13). However, increasing drug resistance, especially rifampicin-resistant (RR)-TB, multidrug-resistant (MDR, resistance to both RIF and INH)-TB, pre-extensively drug resistance [pre-XDR, MDR plus resistance to one fluoroquinolone (FQ)], and XDR-TB [pre-XDR plus additional resistance to one other World Health Organization (WHO) group A drug], threaten TB control (14). In 2021, an estimated 450,000 people developed MDR/RR-TB globally, ~167,000 were diagnosed, and only ~162,000 were initiated on MDR/RR treatment (1). Global treatment success for MDR/RR-TB is only ~60% (1). In MDR/RR-TB, additional resistance to other first-line drugs, such as PZA and EMB, and second-line drugs, including FQ or bedaquiline (BDQ), is problematic as it reduces effective treatment options (15).

In May 2022, the WHO recommended a novel all-oral 6-month regimen of BDQ, preteonamid (Pa), and linezolid (L) plus moxifloxacin (M) (in the absence of FQ resistance) for the treatment of MDR-TB (BPaLM) (16, 17). This new regimen was found to be non-inferior to standard 9- and 12-month MDR-TB regimens and was found to be favorable because of a reduction in adverse events (16, 18). While this new regimen holds great promise, the emergence of FQ and/or BDQ resistance described recently may threaten the longevity of the regimen (19, 20). Thus, understanding the underlying prevalence of FQ/BDQ resistance before introducing the new regimen is crucial (21, 22). Equally important is the ongoing monitoring of resistance development, for example, fostered by the transmission of MTBC strains with particular resistance profiles that can negatively impact diagnostic or treatment strategies in a given geographical region, as shown for the I491F outbreak clone in Eswatini (19, 23, 24). Furthermore, strains of particular MTBC lineages may have minimum inhibitory concentration (MIC) differences to particular drugs, for example, L1 *Mtb* strains have been found to have intrinsically higher Pa MIC compared to L2–L4, and L7 strains (25). Whether or not this is clinically important has not yet been established.

Sierra Leone, a country in West Africa with an estimated TB incidence of 289 per 100,000 population, had a prevalence of MDR/RR-TB of 2.5% and 12% among newly diagnosed and retreatment TB in 2021 (1). Xpert MTB/RIF is the primary molecular TB diagnostic in most hospitals, but smear microscopy continues to be used in primary care clinics. Molecular and phenotypic drug susceptibility testing for all other TB drugs is not performed in the country (26). As such, detailed first- and second-line drug resistance data are unavailable, and the information on phylogeny and transmission dynamics of MDR/RR MTBC strains in the country is very limited.

To close these knowledge gaps, we performed a whole genome sequencing (WGS) study of 238 RR/MDR MTBC strains collected in Sierra Leone between 2016 and 2020. WGS data of the MTBC strains were investigated for phylogenetic classification, resistance prediction, and cluster analysis.

## MATERIALS AND METHODS

### Study design and population

**Fig 1:**
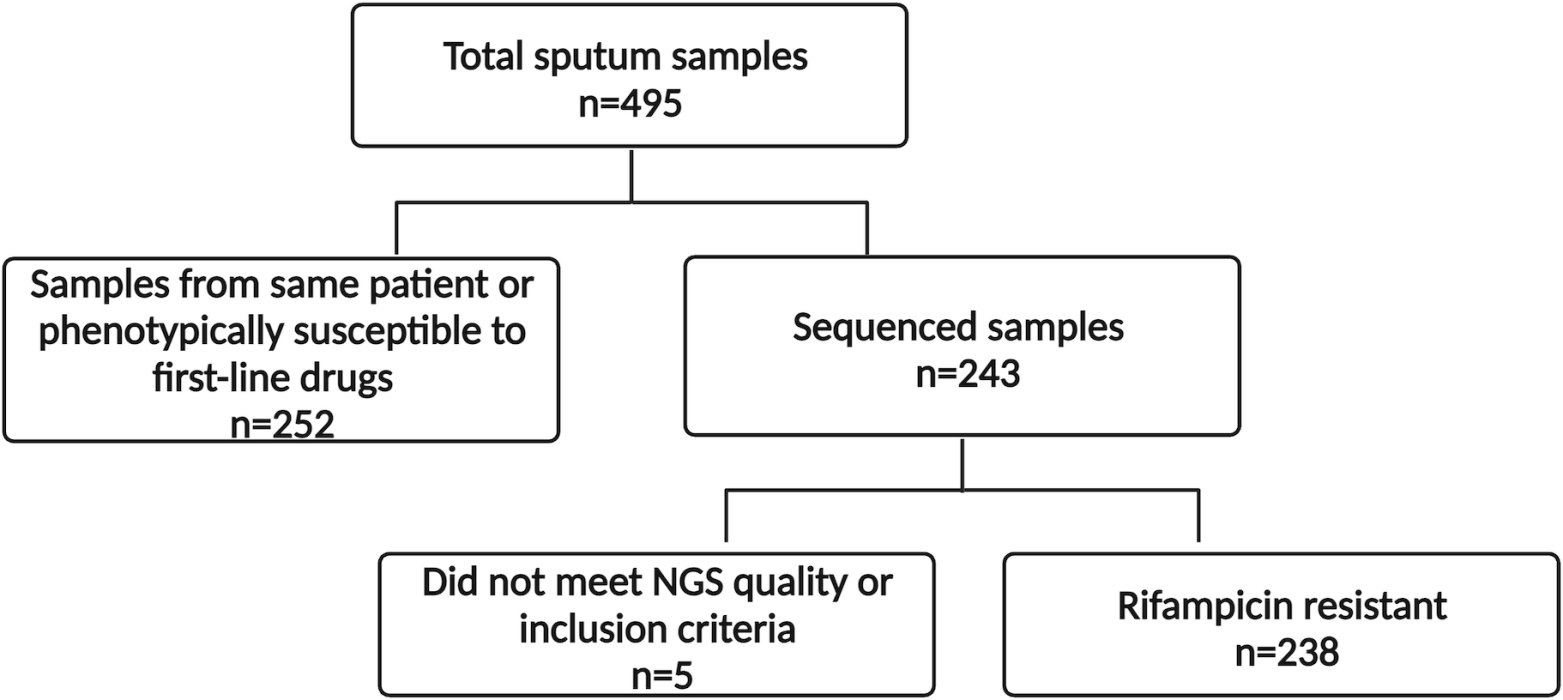
Study design. Inclusion criteria were patient sputum samples positive for *Mycobacterium tuberculosis* complex strains, which were genotypically rifampicin resistant. Only one cultured sample per patient was included in the study and sequenced samples that did not meet NGS quality standards were excluded. *n*, number of samples; NGS, next-generation sequencing. Created with BioRender.

Sputum samples from the National TB Reference Laboratory Freetown, Sierra Leone, of individuals with RR-TB as determined by Xpert MTB/Rif and those with suspected RR-TB who had failed first-line treatment in Sierra Leone between November 2016 and March 2020 were sent to the Supranational Reference Laboratory in Borstel for mycobacterial culture. All RR isolates based on genotypic drug susceptibility tests were included in this analysis. Each strain included in the study came from a different patient. A schematic representation of the study design is shown in Fig. 1.

### Whole genome sequencing and analysis

The DNA of MTBC strains was extracted as previously described (27). The genomes were sequenced and analyzed with bioinformatics pipelines as described by Grobbel et al. (28) and Merker et al. (29). Briefly, genomic DNA was sequenced using Illumina Technology (NextSeq 500 or MiSeq) using the Baym protocol (30) and Nextera library preparation kits following the manufacturer’s instructions (Illumina, San Diego, CA). The FASTQ data were analyzed with the MTBseq pipeline (31). Briefly, reads were mapped to the H37Rv genome [GenBank ID: NC_000962.3 (32)] with BWA (33), processed with SAMtools (34), and the mappings were refined with the GATK 3.8 (35).

For variant detection, SAMtools (34)-derived mpileup files were filtered for minimum thresholds of at least two reads indicating a variant in both forward and reversed orientation, two reads calling the allele with at least a phred score of 4, and 5% allele frequency for resistance determination, and for phylogeny, four reads mapped in each forward and reverse direction, respectively, with 75% allele frequency and at least four calls with a phred score of at least 20.

Genomic SNP positions with a reliable base call in at least 95% of the strains and covered in all strains were concatenated to a sequence alignment. SNPs within a window of 12 bp from each other and those located in repetitive regions or resistance-associated genes were excluded to avoid calling SNPs related to insertions and deletions artifacts (29). Strains were phylogenetically classified, and transmission clusters were inferred with single linkage clustering using distance cutoffs of ≤5 and ≤12 SNPs. Raw FASTQs were uploaded to the European Nucleotide Archive (Table S1).

### Resistance analysis

Genotypic resistance predictions were based on a curated Research Center Borstel mutation catalog, as described by Grobbel et al. (28), and the WHO’s catalog (36). Mutations linked to phenotypic drug resistance were marked as resistant, and unclear ones were classified as not resistant. When no mutation was detected, strains were defined as susceptible. Drug-resistant types were classified based on the WHO’s classification (14).

### Phylogenomic analysis

A maximum parsimony tree (MPT) was built with the software BioNumerics version 7.6.3 (Biomerieux) from the aligned sequences of concatenated SNPs. Numbers on branches indicate the number of distinct SNP positions between isolates. Using distinct SNP sites, maximum likelihood trees (MLTs) were calculated with IQ-TREE 2, an efficient method for phylogenetic inference (37), using ModelFinder (38), an automated model selection, including ascertainment bias correction, and ultrafast bootstrap approximation (39) with resampling of 1,000. The MLT was midpoint rooted with the FigTree software version 1.4.4. The trees were visualized with Interactive Tree of Life (iTOL) 5.7 (40).

### Statistical analysis

Descriptive statistics were performed (distribution frequency) and graphs were drawn with R software version 4.2.1.

## RESULTS

### Study population

A total of 238 MTBC strains were included in the study (Table 1), 63% (*n* = 151), and they were from retreatment patients. The majority of strains were obtained from men (43%, *n* = 102), and the median age was 34 years (interquartile range 30–39). Among patients with known HIV status, 15% (25/162) were HIV positive.

**TABLE 1:** Main demographic characteristics of study participants^*a*^

		*n* (RR/MDR)	(%)
Total		238	
Sex	Female	41	17
	Male	102	43
	Unknown	95	40
Age range (years)	≤19	13	6
	20–29	50	21
	30–39	48	20
	40–49	37	16
	50–59	20	8
	≥60	8	3
	Unknown	62	26
Type	New	10	4
	Retreatment	151	63
	Unknown	77	32
HIV status	Negative	137	58
	Positive	25	11
	Unknown	76	32

^*a*^ HIV, human immunodeficiency virus; MDR, multidrug-resistant; RR, rifampicin-resistant.

### MTBC population structure

**Fig 2:**
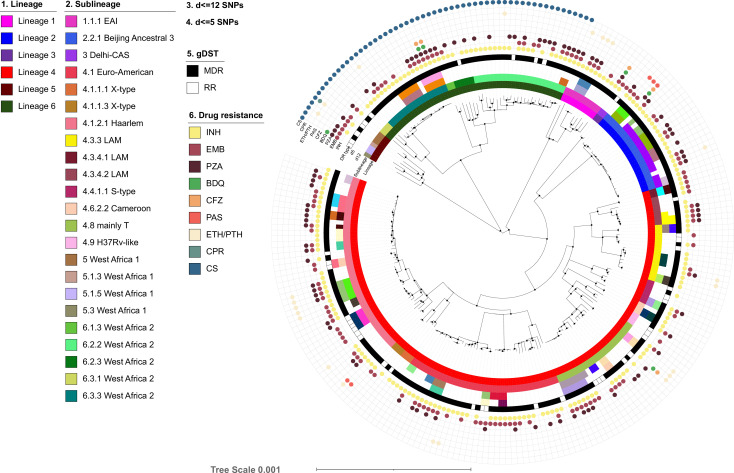
Phylogeny, lineage classification, and drug resistance patterns of the *Mycobacterium tuberculosis* complex strains from Sierra Leone. The maximum likelihood tree was based on a concatenated single-nucleotide polymorphism (SNP) alignment of 328 *M. tuberculosis* complex (MTBC) strains. The alignment was based on 15,089 informative SNP sites. Ultrafast bootstrap values (+0.95) are indicated on the tree branches with black dots and the circles from the inner ring to the outer show: 1. The seven main MTBC lineages; 2. the MTBC sublineages; 3. and 4. show the genome clusters based on strain SNP distances of ≤12 SNPs and ≤5 SNPs, respectively (strains sharing the same color belong to the same cluster), 5. genotypic drug susceptibility types (gDST); and 6. the colored dots indicate genotypic resistance to the respective drug. INH, isoniazid; EMB, ethambutol; PZA, pyrazinamide; PAS, para-aminosalicylic acid; CS, cycloserine; ETH, ethionamide; PTH, proteonamid; CFZ, clofazimine; BDQ, bedaquiline; CPR, capreomycin; MDR, multidrug-resistant (resistance to at least isoniazid and rifampicin); RR, rifampicin-resistant.

WGS data analysis revealed 15,089 informative SNPs differentiating any of the 238 MTBC strains. These SNPs were used to calculate a maximum likelihood phylogeny based on a concatenated SNP alignment (Fig. 2). Based on canonical SNP signatures (12), the 238 strains were classified into six main MTBC lineages (L1–L6) (Fig. 2; Fig. S1; Table 2). Strains of L4 were most frequent (*n* = 147, 62%), followed by strains of L6 (*Maf, n* = 50, 21%), L2 (*n* = 22, 9%), L1 (*n* = 10, 4%), L5 (*Maf, n* = 7, 3%), and L3 (*n* = 2, 0.8%). The strains were further categorized into sub-lineages as follows: 4.1.2.1 Haarlem (*n* = 45, 19%), 4.1 Euro-American (*n* = 38, 16%), 6.2.2 West Africa 2 and 4.8 mainly T (*n* = 23, 9.7%), and 2.2.1 Beijing Ancestral 3 (*n* = 22, 9.2%) (Fig. 2; Tables S1 and S2.1).

**TABLE 2:** MTBC strain proportions, clustering, and cluster rate within the lineages

Main lineages	∑ no. of strains (%)	# Clustered	Cluster rate
Lineage 1	10 (4.2)	3	30
Lineage 2	22 (9.2)	19	86
Lineage 3	2 (0.8)	0	0
Lineage 4	147 (61.8)	70	48
Lineage 5	7 (2.9)	0	0
Lineage 6	50 (21)	12	24
Total	238	104	44

### Drug resistance

**Fig 3:**
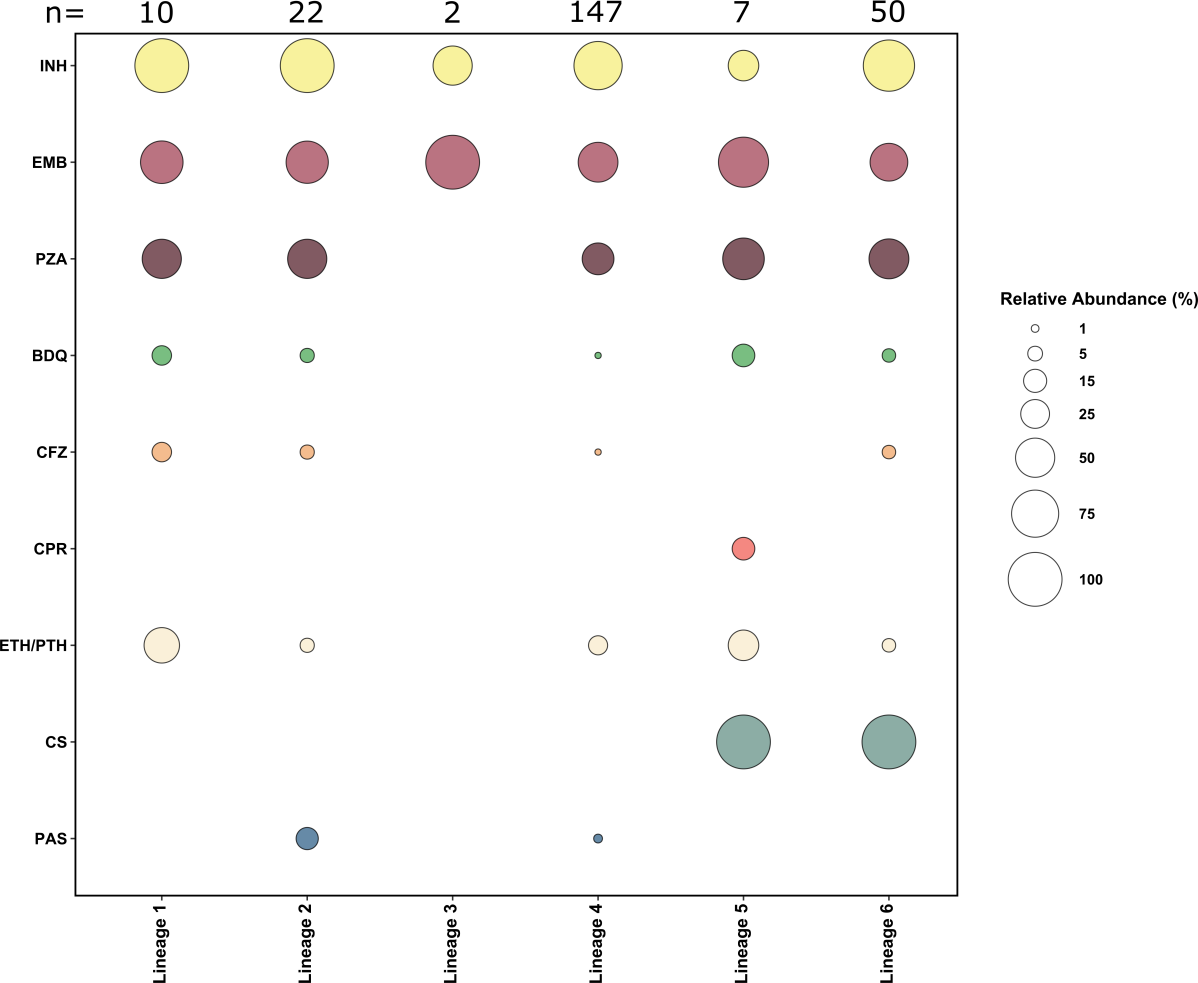
Drug resistance to first- and second-line anti-tuberculosis drugs per lineage of *Mycobacterium tuberculosis* complex strains investigated. The bubble plot shows the relative abundance of *M. tuberculosis* complex (MTBC) strains for each lineage that developed resistance to the corresponding drug. On the left *y*-axis are the abbreviated names of drugs: INH, isoniazid; EMB, ethambutol; PZA, pyrazinamide; CS, cycloserine; ETH, ethionamide; PTH, proteonamid; PAS, para-aminosalicylic acid; CFZ, clofazimine; BDQ, bedaquiline; and CPR, capreomycin. On the *x*-axis at the bottom are the MTBC strain lineages and indicated at the top are the total number of MTBC strains per lineage. *n* = number of isolates

We then performed a genotypic resistance prediction based on high-confidence resistance mutations. Overall, resistance prevalence was 82% (*n* = 196), 53% (*n* = 126), and 39% (*n* = 92) for INH, EMB, and PZA, respectively (Fig. 3; Fig. S2–S6; Table S2.2). No resistance to FQ and linezolid was detected; however, five strains were resistant to BDQ/clofazimine (CFZ) based on mutations in *Rv0678* (Fig. S4). A total of 61 (26%) strains were found to be resistant to all first-line drugs; three had additional resistance to BDQ/CFZ (Table S2.3). Resistance patterns were similar across strains from all lineages, except for cycloserine (CS), as all L5 and L6 strains were inherently resistant to CS (Fig. 3). Out of the five BDQ/CFZ resistant strains, one belonged to L1.1.1, one to L4.8, one to L5, and two to L6 (Table S2.4).

All the RIF resistance-conferring mutations were found in the *rpoB* gene. The most prevalent mutation was *rpoB* S450L (41%, *n* = 98), which is part of the WHO group_1 mutations (2001) associated with resistance (36) (Table S3.1). The *rpoB* S450L mutation was found in strains of all lineages except L2 ( Table S4). Despite this, there was a diversity of *rpoB* mutations, with 32 (13%) of the strains having one of the so-called RIF-borderline mutations (*rpoB* D435Y, L452P, H445L/N, and L430P) (41, 42). Such strains may test phenotypically susceptible to RIF. Also, three strains had the *rpoB* I491V and V170F resistance mutations, which are found outside of the RIF-resistance-determining region (RRDR) (23, 24, 36, 43) (Tables S1, S3.1, and S4), and thus, not detected by commercial molecular RR tests such as Xpert MTB/RIF assay.

Mutations linked with resistance to INH were found in the *katG* gene and the *fabG1-inhA* regulatory region (Tables S1, S3.2, and S5) (44). The *katG* S315T resistance mutation was most prevalent (*n* = 133, 56%) and found in strains of all lineages except L3 and L5 (Tables S1, S3.2, and S5). Besides, 53 strains had mutations in *fabG1-inhA/inhA*, out of which 37 acquired a second mutation in *katG* (Tables S3.2 and S5). Of those, the most prevalent mutation was *fabG1* 15t>c (18 strains), which is classified as WHO group_2 borderline mutation; 12 did not have any additional INH mutations, and 4 had an additional *katG* S315T mutation (Tables S1 and S3.2). Also, of note were the 12 strains with *fabG1* L203L mutations; however, 10 of these strains had also developed the higher level *katG* S315T resistance mutation (Tables S1, S3.2, and S5).

### Transmission (genomic clustering)

**Fig 4:**
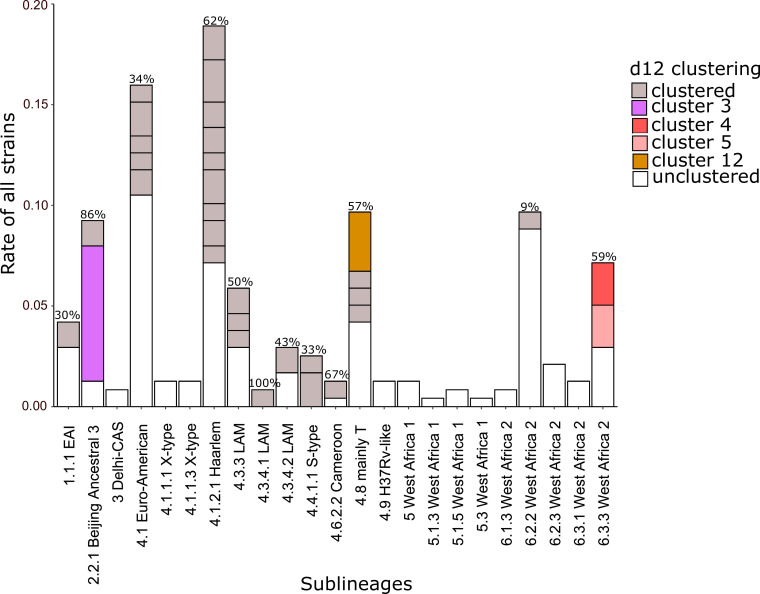
Clustering proportions of *Mycobacterium tuberculosis* complex strains by sublineage. The bar plot shows the distribution of *M. tuberculosis* complex strains that clustered versus unclustered based on sublineages. The total cluster rate per sublineage is indicated on the top of the bars and distinct clusters are separated by lines. Large clusters with five or more strains are presented in specific colors, while gray represents clusters with less than five strains and white represents the proportion of unclustered strains.

**Fig 5:**
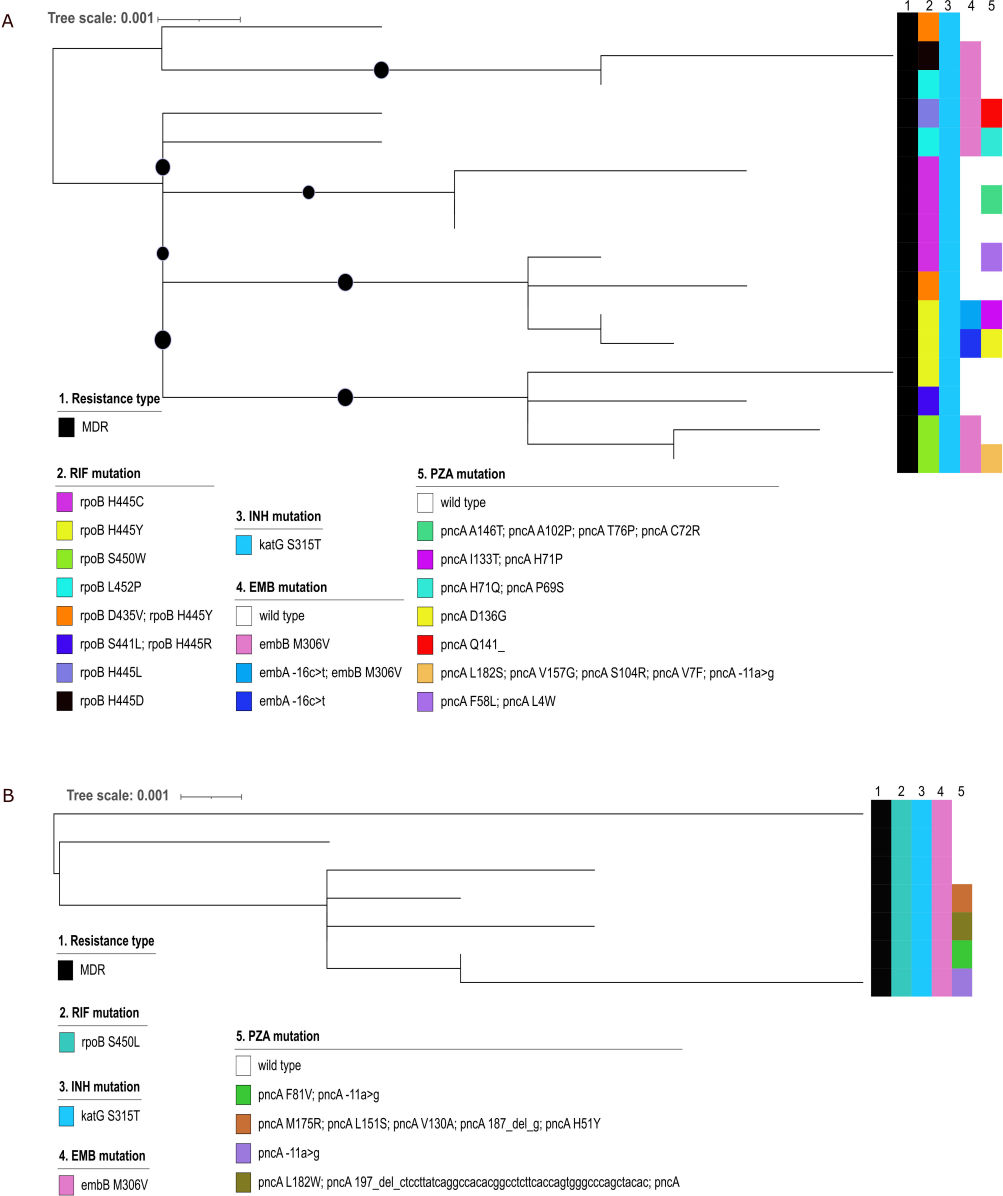
Phylogeny of the strains of the two largest clusters of multidrug-resistant *Mycobacterium tuberculosis* strains. Maximum likelihood trees were calculated for the *M. tuberculosis* strains of the two largest multidrug-resistant clusters identified in the study. (**A**) The midpoint-rooted phylogeny of the 16 clustered *M. tuberculosis* strains of "cluster 3" belonging to the sublineage 2.2.1 Beijing Ancestral 3. The alignment was based on 1,174 informative SNP sites. This tree shows the transmission of an isoniazid-resistant clone with multiple independent developments to multidrug resistance (MDR—resistance to at least isoniazid and rifampicin), shown by the different rifampicin resistance mutations. The strains also had developed different ethambutol and pyrazinamide resistance mutations. (**B**) The midpoint-rooted phylogeny of the seven clustered *M. tuberculosis* strains of "cluster 12" belonging to the sub-lineage 4.8 mainly T. The alignment was based on 466 informative SNP sites. In this tree, a single MDR clone was transmitted, which was also resistant to ethambutol, shown by the same resistance mutation for all the seven strains. However, the strains developed different pyrazinamide resistance mutations. 1. The genotypic drug resistance type; 2–5. the color-coded resistance mutations of the first-line drugs identified for the respective drugs; and the black dots on the tree branches indicate the ultrafast bootstrap values (+0.95). RIF, rifampicin; INH, isoniazid; EMB, ethambutol; PZA, pyrazinamide; MDR, multidrug-resistant.

Using a maximum distance of 12 SNPs between two strains to define a cluster, 104 (44%) strains were grouped into 31 clusters ranging in size from 2 to 16 strains (Fig. 4; Fig. S7; Table 2). We linked the cluster data with resistance mutation profiles to better understand the transmission dynamics of RR/MDR strains. Indeed, while all strains of the largest cluster group_3 shared the *katG* S315T mutation (Fig. 5A) (16 isolates of 2.2.1 Beijing sublineage), they had different *rpoB*, *embB*, and *pncA* resistance mutations, subdividing the clustered strains into sub-clusters as indicated in the MPT (Fig. S8). The second-largest cluster group_13 consisted of seven 4.8 mainly T sublineage MDR strains (Fig. 5B); all strains shared the same RIF *rpoB* S450L, INH *katG* S315T, and EMB *embB* M306V resistance mutations indicating the ongoing spread of this MDR strain in the country (Fig. 5B).

**Fig 6:**
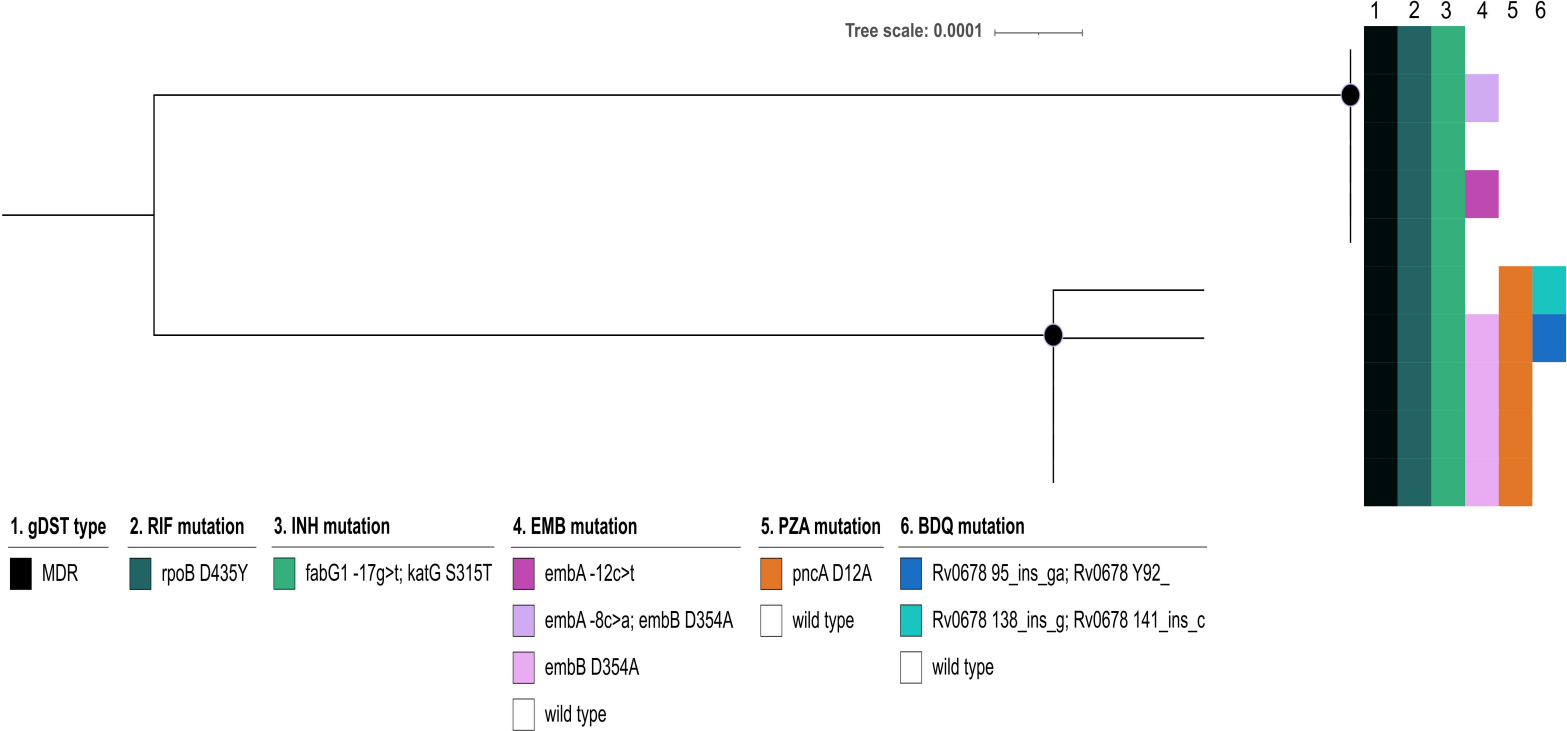
Phylogeny of the largest cluster of endemic multidrug-resistant *Mycobacterium africanum* strains. The mid-point-rooted maximum likelihood tree was calculated for the 10 multidrug-resistant (MDR, resistance to at least isoniazid and rifampicin) *M. africanum* strains belonging to sublineage 6.3.3 West Africa 2. The alignment was based on 1,924 informative SNP sites. The tree shows a single sublineage 6.3.3 West Africa 2 MDR clone transmitting, which formed two distinct clusters (clusters 4 and 5). 1. The genotypic drug susceptibility type (gDST); 2–6. are the color-coded resistance mutations of the first-line drugs identified for the respective drugs; and the black dots on the tree branches indicate the ultrafast bootstrap values (+0.95). RIF, rifampicin; INH, isoniazid; EMB, ethambutol; PZA, pyrazinamide; BDQ, bedaquiline; MDR, multidrug-resistant.

Clustering also indicated smaller transmission events of RR/MDR L6 strains (Fig. 6). Out of the 51 L6 strains, 12 (24%) clustered (Tables S1 and S2.5). Of the 6.3.3 West Africa 2 sublineage strains, 10 out of 17 (59%) were clustered, forming two clusters (group_4 and group_5) of five strains each (Fig. 6). Interestingly, while the cluster analysis separated the strains into two clusters, they were closely related in the phylogeny and share the RIF *rpoB* D435Y mutation and INH *fabG1*-17g>t/*katG* S315T double mutation, indicating the emergence from a common MDR ancestor (Fig. 6). However, only the strains of group_5 developed further resistance to EMB and PZA, rendering them fully first-line resistant (Fig. 6). Also, two of the strains developed BDQ/CFZ resistance due to mutations in *Rv0678* (Table S1).

## DISCUSSION

In this study, we employed WGS to characterize RR/MDR MTBC strains from Sierra Leone. We found that one in five strains investigated was resistant to all first-line drugs. While no resistance to FQ was observed, five strains had resistance to BDQ/CFZ due to *Rv0678* mutations. The cluster rate exceeded 40%, indicating ongoing transmission of RR/MDR TB, which contributes significantly to the disease burden in the country. Strikingly, *Maf* L6 strains constituted 21% of the MDR MTBC strains analyzed and formed a longitudinal outbreak with two branches, each constituting five strains. Within one of these branches, all strains were resistant to all first-line drugs, and two strains had developed additional BDQ/CFZ resistance. There was a high diversity of drug resistance mutations with a significant number of so-called borderline INH and RIF resistance mutations, potentially allowing the use of these first-line drugs albeit with higher dosing.

None of the investigated MTBC strains had FQ resistance, which contrasts with data from other West African countries. For instance, in Nigeria, FQ resistance was 13% (17/132 of RR-TB) in a study conducted between 2018 and 2020 (45), while in Ghana, 36% (11/31) of MDR-TB strains were FQ resistant (46).

We found five MTBC strains (2%) with resistance to BDQ and CFZ due to mutations in *Rv0678*, a transcriptional repressor of the MmpS5–MmpL5 efflux pump (47, 48). Mutations in *Rv0678* are the primary drivers of BDQ/CFZ resistance in clinical strains in South Africa (20) and Moldova (21). Also, these mutations are linked to treatment failure and can potentially cause an increase in BDQ/CFZ resistance over a short timeframe (20).

Considering the low prevalence of BDQ resistance (2%) and the absence of FQ resistance in Sierra Leone, both the 9–12-month all-oral regimens and the new 6-month BPaLM are likely to be effective treatment regimens for most patients (16, 17). BDQ/CFZ resistance may emerge without direct drug exposure (23, 24, 43, 49), and since the exact mechanism is yet to be identified, it is crucial to prioritize prospective surveillance for BDQ/CFZ resistance and mechanisms involved in selecting MTBC strains with *Rv0678* mutations. This is especially significant as BDQ is an essential drug in the new BPaLM regimen and the rapid development of resistance may reduce the efficacy of the regimen (50).

Our investigation of the MTBC strain diversity identified strains of six main MTBC lineages circulating and causing RR/MDR-TB in Sierra Leone. Overall, the widely distributed and diverse L4 strains were the most prevalent (51). L4 strains are highly successful globally due to their genotypic and phenotypic diversity (51–53). The high proportion of L4 strains in this study linked to a cluster rate of 62% is in line with previous studies (2, 51). Lineage 2 strains were the third most prevalent strains; surprisingly, they all belonged to the 2.2.1 Beijing Ancestral 3 sublineage, also showing a high cluster rate of 86%, indicating effective MDR-TB transmission. Recent studies, for example, from Eastern European countries, have found associations between modern L2 strains such as L2.2.3 (54) and L2.2.1 (55) with high rates of clustering and transmission (56–58). Studies from India and South Africa indicate that strains of ancestral Beijing lineages may also develop high drug resistance rates linked with ongoing transmission of few MDR/pre-XDR/XDR strains in a given setting (19, 59). The emergence of ancestral Beijing strains in Sierra Leone warrants close monitoring and further investigations towards cross-border spread in Africa, introduction by migration followed by local spread, and the overall importance of this strain type for the MDR epidemic.

In contrast to other geographical regions, 24% of strains belonged to *Maf*, the majority were L6 (classified as West African 2), confirming the findings from another study from West Africa (60). All *Maf* strains were resistant to CS, a drug classified as group B by the WHO because of 1 bp frameshift deletion in *ald,* as described previously (61).

In line with findings from other African countries such as Namibia, we found a high diversity of INH/RIF resistance mutations with a substantial proportion of strains having so-called borderline resistance mutation in *rpoB* and/or in the *inhA-fabG1* promotor region, which are difficult to detect by phenotypic assays (41, 42, 62). In patients infected with MTBC strains with lower-level resistance mutations, high-dose INH and/or RIF may overcome resistance, presenting a viable treatment option, especially for patients with MDR/pre-XDR/XDR TB with advanced resistance patterns (63, 64). However, host genetic factors that lead to enhanced drug metabolisms and/or reduced bioavailability need to be investigated as they are likely to contribute to the higher frequency of strains with borderline resistance mutations in the region (65–67). High-dose regimens can only be applied when both pathogen and host genetics/phenotypes are available.

Still, the high prevalence of MTBC strains in Sierra Leone and Namibia with RR/MDR-TB patients underlines their significance for the drug resistance TB epidemiology. Moreover, since strains with borderline INH resistance mutations can also develop higher-level resistance by a second mutation (42), it is, therefore, essential to have molecular methods for resistance detection to keep track of low-level resistance mutations to prevent misclassification of resistant strains and reduce treatment failure.

We detected three strains with resistance mutations outside the rifampicin RRDR (*rpoB* I491V and V170F), which are not detected by commercial molecular RR tests such as Xpert MTB/RIF assay (23, 24, 43). Consequently, patients are usually treated with drug-susceptible regimens resulting in higher rates of treatment failures and possibly enhanced transmission. Such strains have also proven to be challenging for severe MDR-TB control in other African countries such as Eswatini (23).

### Conclusion

Our data indicate that resistance to group A, B, and C MDR-TB treatment drugs is limited in Sierra Leone. Hence, the short all-oral-6-month BPaLM or the recently proposed 6–9-month or 9–12-month regimens offer great potential to treat most of the MDR-TB cases in the country successfully. Our data on the MTBC strains' population structure point toward the potential importance of ancestral Beijing strains for the MDR-TB epidemic in Africa. We also demonstrated that MDR *Maf* strains contribute significantly to the MDR-TB burden in the country.

### Limitations

Our study was limited by the small sample size and lack of metadata to help make associations among the isolates. However, the thorough genotypic testing and in-depth analysis revealed noteworthy findings.

## SUPPLEMENTAL MATERIAL

Tables S4 and S5 spectrum.02405-23-s0001.docx Supplementary tables.

Figures S5, S6, S7, S8 spectrum.02405-23-s0002.pdf Supplementary figures.

Figures S1, S2, S3, S4 spectrum.02405-23-s0003.pdf Supplementary figures.

Table S1 spectrum.02405-23-s0004.csv Supplementary table.

Table S2 spectrum.02405-23-s0005.docx Supplementary table.

Table S3 spectrum.02405-23-s0006.docx Supplementary table.

ASM does not own the copyrights to Supplemental Material that may be linked to, or accessed through, an article. The authors have granted ASM a non-exclusive, world-wide license to publish the Supplemental Material files. Please contact the corresponding author directly for reuse.
