# Supplementary material for: Molecular determinants of multidrug-resistant tuberculosis in Sierra Leone
Source: Microbiol Spectr. 2024 Jan 30;12(3):e02405-23. doi: 10.1128/spectrum.02405-23 (PMC10923214; doi:10.1128/spectrum.02405-23)
Supplement: Tables S4 and S5 — Supplementary tables. [file spectrum.02405-23-s0001.docx]

Supplementary table S4 and 5

Supplementary table S4: Distribution of Rifampicin resistance mutations per lineage

| **RMP resist mutations** | **Lineage 1** | **%** | **Lineage 2** | **%** | **Lineage 3** | **%** | **Lineage 4** | **%** | **Lineage 5** | **%** | **Lineage 6** | **%** | **Grand Total** |
| --- | --- | --- | --- | --- | --- | --- | --- | --- | --- | --- | --- | --- | --- |
| rpoB 1277_del_gcacca | 0 | 0 | 0 | 0 | 0 | 0 | 0 | 0 | 1 | 100 | 0 | 0 | 1 |
| rpoB 1277_del_gcacca; rpoB S450L | 0 | 0 | 0 | 0 | 0 | 0 | 1 | 100 | 0 | 0 | 0 | 0 | 1 |
| rpoB 1288_del_ctgagccaa | 0 | 0 | 0 | 0 | 0 | 0 | 0 | 0 | 0 | 0 | 1 | 100 | 1 |
| rpoB 1291_ins_gcc | 0 | 0 | 0 | 0 | 0 | 0 | 1 | 100 | 0 | 0 | 0 | 0 | 1 |
| rpoB 1295_del_aattcatgg | 0 | 0 | 0 | 0 | 0 | 0 | 0 | 0 | 0 | 0 | 1 | 100 | 1 |
| rpoB 1296_ins_ttc | 0 | 0 | 0 | 0 | 0 | 0 | 0 | 0 | 0 | 0 | 1 | 100 | 1 |
| rpoB 1304_del_accaga | 0 | 0 | 0 | 0 | 0 | 0 | 1 | 100 | 0 | 0 | 0 | 0 | 1 |
| rpoB 1307_del_aga | 0 | 0 | 0 | 0 | 0 | 0 | 0 | 0 | 0 | 0 | 1 | 100 | 1 |
| rpoB 1309_del_aac | 0 | 0 | 0 | 0 | 0 | 0 | 1 | 50 | 0 | 0 | 1 | 50 | 2 |
| rpoB 1309_del_aac; rpoB S450L | 0 | 0 | 0 | 0 | 0 | 0 | 1 | 100 | 0 | 0 | 0 | 0 | 1 |
| rpoB D435F | 1 | 25 | 0 | 0 | 0 | 0 | 0 | 0 | 0 | 0 | 3 | 75 | 4 |
| rpoB D435L | 0 | 0 | 0 | 0 | 0 | 0 | 2 | 100 | 0 | 0 | 0 | 0 | 2 |
| rpoB D435V | 2 | 8 | 0 | 0 | 0 | 0 | 18 | 72 | 2 | 8 | 3 | 12 | 25 |
| rpoB D435V; rpoB 1307_del_aga | 0 | 0 | 0 | 0 | 0 | 0 | 0 | 0 | 0 | 0 | 1 | 100 | 1 |
| rpoB D435V; rpoB H445Y | 0 | 0 | 2 | ## | 0 | 0 | 0 | 0 | 0 | 0 | 0 | 0 | 2 |
| rpoB D435V; rpoB S450L | 0 | 0 | 0 | 0 | 0 | 0 | 2 | 100 | 0 | 0 | 0 | 0 | 2 |
| rpoB D435Y | 0 | 0 | 0 | 0 | 0 | 0 | 3 | 23 | 0 | 0 | 10 | 77 | 13 |
| rpoB D435Y; rpoB I491L | 0 | 0 | 0 | 0 | 0 | 0 | 1 | 100 | 0 | 0 | 0 | 0 | 1 |
| rpoB D435Y; rpoB L452M | 0 | 0 | 0 | 0 | 0 | 0 | 1 | 100 | 0 | 0 | 0 | 0 | 1 |
| rpoB D435Y; rpoB N437H | 0 | 0 | 0 | 0 | 0 | 0 | 0 | 0 | 0 | 0 | 1 | 100 | 1 |
| rpoB D435Y; rpoB S441L; rpoB H445Y | 0 | 0 | 0 | 0 | 0 | 0 | 0 | 0 | 0 | 0 | 1 | 100 | 1 |
| rpoB H445C | 0 | 0 | 3 | 60 | 0 | 0 | 2 | 40 | 0 | 0 | 0 | 0 | 5 |
| rpoB H445D | 0 | 0 | 1 | 11 | 0 | 0 | 7 | 78 | 1 | 11.1 | 0 | 0 | 9 |
| rpoB H445D; rpoB L452P | 0 | 0 | 0 | 0 | 0 | 0 | 1 | 100 | 0 | 0 | 0 | 0 | 1 |
| rpoB H445L | 0 | 0 | 2 | 33 | 0 | 0 | 3 | 50 | 0 | 0 | 1 | 17 | 6 |
| rpoB H445N | 0 | 0 | 0 | 0 | 0 | 0 | 1 | 50 | 1 | 50 | 0 | 0 | 2 |
| rpoB H445P | 1 | 100 | 0 | 0 | 0 | 0 | 0 | 0 | 0 | 0 | 0 | 0 | 1 |
| rpoB H445R | 3 | 50 | 2 | 33 | 0 | 0 | 1 | 17 | 0 | 0 | 0 | 0 | 6 |
| rpoB H445Y | 0 | 0 | 4 | 27 | 0 | 0 | 7 | 47 | 0 | 0 | 4 | 27 | 15 |
| rpoB L430P | 0 | 0 | 1 | 33 | 0 | 0 | 2 | 67 | 0 | 0 | 0 | 0 | 3 |
| rpoB L452P | 0 | 0 | 4 | 50 | 0 | 0 | 1 | 13 | 0 | 0 | 3 | 38 | 8 |
| rpoB Q429H; rpoB L430P; rpoB D435Y; rpoB H445Q | 0 | 0 | 0 | 0 | 0 | 0 | 0 | 0 | 0 | 0 | 1 | 100 | 1 |
| rpoB Q429L; rpoB D435Y; rpoB N437Y | 0 | 0 | 0 | 0 | 0 | 0 | 1 | 100 | 0 | 0 | 0 | 0 | 1 |
| rpoB Q432E | 0 | 0 | 0 | 0 | 0 | 0 | 1 | 100 | 0 | 0 | 0 | 0 | 1 |
| rpoB Q432K | 0 | 0 | 0 | 0 | 0 | 0 | 2 | 100 | 0 | 0 | 0 | 0 | 2 |
| rpoB Q432K; rpoB S450L | 0 | 0 | 0 | 0 | 0 | 0 | 0 | 0 | 0 | 0 | 1 | 100 | 1 |
| rpoB Q432L | 0 | 0 | 0 | 0 | 0 | 0 | 1 | 100 | 0 | 0 | 0 | 0 | 1 |
| rpoB S431R; rpoB S450W | 0 | 0 | 1 | ## | 0 | 0 | 0 | 0 | 0 | 0 | 0 | 0 | 1 |
| rpoB S441L | 0 | 0 | 0 | 0 | 0 | 0 | 1 | 100 | 0 | 0 | 0 | 0 | 1 |
| rpoB S441L; rpoB H445R | 0 | 0 | 1 | ## | 0 | 0 | 0 | 0 | 0 | 0 | 0 | 0 | 1 |
| rpoB S441L; rpoB S450L | 0 | 0 | 0 | 0 | 0 | 0 | 1 | 100 | 0 | 0 | 0 | 0 | 1 |
| rpoB S450F | 0 | 0 | 0 | 0 | 0 | 0 | 1 | 100 | 0 | 0 | 0 | 0 | 1 |
| rpoB S450L | 3 | 3.19 | 0 | 0 | 2 | 2.13 | 74 | 79 | 1 | 1.06 | 14 | 15 | 94 |
| rpoB S450L; rpoB I491V | 0 | 0 | 0 | 0 | 0 | 0 | 0 | 0 | 1 | 100 | 0 | 0 | 1 |
| rpoB S450Q | 0 | 0 | 0 | 0 | 0 | 0 | 2 | 100 | 0 | 0 | 0 | 0 | 2 |
| rpoB S450W | 0 | 0 | 1 | 14 | 0 | 0 | 4 | 57 | 0 | 0 | 2 | 29 | 7 |
| rpoB V170F | 0 | 0 | 0 | 0 | 0 | 0 | 1 | 100 | 0 | 0 | 0 | 0 | 1 |
| rpoB V170F; rpoB M434I; rpoB H445N; rpoB L452V | 0 | 0 | 0 | 0 | 0 | 0 | 1 | 100 | 0 | 0 | 0 | 0 | 1 |
| Total | 10 |  | 22 |  | 2 |  | 147 |  | 7 |  | 50 |  | 238 |
|  |  |  |  |  |  |  |  |  |  |  |  |  |  |
| mutations outside RRDR colored in red | |  |  |  |  |  |  |  |  |  |  |  |  |
| borderline mutations colored in purple | |  |  |  |  |  |  |  |  |  |  |  |  |

Supplementary table S5. Frequency of isoniazid resistance mutations per lineage

| **INH resist mutations** | **Lineage 1** | **%** | **Lineage 2** | **%** | **Lineage 3** | **%** | **Lineage 4** | **%** | **Lineage 5** | **%** | **Lineage 6** | **%** | **Grand Total** |
| --- | --- | --- | --- | --- | --- | --- | --- | --- | --- | --- | --- | --- | --- |
| fabG1 -15c>t | 0 | 0 | 0 | 0 | 0 | 0 | 10 | 83.3 | 1 | 8.3 | 1 | 8.3 | 12 |
| fabG1 -15c>t; katG 63_del_c; katG 36_del_c | 0 | 0 | 0 | 0 | 0 | 0 | 1 | 100 | 0 | 0 | 0 | 0 | 1 |
| fabG1 -15c>t; **katG S315T** | 4 | 100 | 0 | 0 | 0 | 0 |  | 0 | 0 | 0 | 0 | 0 | 4 |
| fabG1 -15c>t; katG W191R | 0 | 0 | 0 | 0 | 0 | 0 | 1 | 100 | 0 | 0 | 0 | 0 | 1 |
| fabG1 -17g>t; fabG1 -8t>c; **katG S315T** | 0 | 0 | 0 | 0 | 0 | 0 |  | 0 | 0 | 0 | 1 | 100 | 1 |
| fabG1 -17g>t; **katG S315T** | 0 | 0 | 0 | 0 | 0 | 0 | 3 | 21.4 | 0 | 0 | 11 | 79 | 14 |
| fabG1 -17g>t; katG Y98C | 0 | 0 | 0 | 0 | 0 | 0 |  | 0 | 0 | 0 | 1 | 100 | 1 |
| fabG1 -8t>a; **katG S315T** | 0 | 0 | 0 | 0 | 0 | 0 | 1 | 100 | 0 | 0 | 0 | 0 | 1 |
| fabG1 -8t>c | 0 | 0 | 0 | 0 | 0 | 0 | 2 | 100 | 0 | 0 | 0 | 0 | 2 |
| fabG1 -8t>c; **katG S315T** | 0 | 0 | 3 | 100 | 0 | 0 |  | 0 | 0 | 0 | 0 | 0 | 3 |
| fabG1 L203L | 0 | 0 | 0 | 0 | 0 | 0 | 2 | 100 | 0 | 0 | 0 | 0 | 2 |
| fabG1 L203L; **katG S315T** | 0 | 0 | 0 | 0 | 0 | 0 | 10 | 100 | 0 | 0 | 0 | 0 | 10 |
| inhA S94A | 0 | 0 | 0 | 0 | 0 | 0 |  | 0 | 1 | 100 | 0 | 0 | 1 |
| katG 1003_ins_g | 0 | 0 | 0 | 0 | 0 | 0 | 1 | 50 | 0 | 0 | 1 | 50 | 2 |
| katG 1079_del_gg | 0 | 0 | 0 | 0 | 0 | 0 | 1 | 100 | 0 | 0 | 0 | 0 | 1 |
| katG 1253_del_gc | 0 | 0 | 0 | 0 | 0 | 0 | 1 | 100 | 0 | 0 | 0 | 0 | 1 |
| katG 1335_ins_t | 0 | 0 | 0 | 0 | 0 | 0 |  | 0 | 0 | 0 | 1 | 100 | 1 |
| katG 1432_del_g | 0 | 0 | 0 | 0 | 0 | 0 |  | 0 | 0 | 0 | 1 | 100 | 1 |
| katG 1745_ins_g | 0 | 0 | 0 | 0 | 0 | 0 |  | 0 | 0 | 0 | 1 | 100 | 1 |
| katG 1861_del_gtgactcgcatt | 0 | 0 | 0 | 0 | 0 | 0 | 1 | 100 | 0 | 0 | 0 | 0 | 1 |
| katG 2010_del_c | 0 | 0 | 0 | 0 | 0 | 0 | 1 | 100 | 0 | 0 | 0 | 0 | 1 |
| katG 2087_ins_c | 0 | 0 | 0 | 0 | 0 | 0 |  | 0 | 0 | 0 | 1 | 100 | 1 |
| katG 23_ins_a | 0 | 0 | 0 | 0 | 0 | 0 |  | 0 | 0 | 0 | 1 | 100 | 1 |
| katG 29_ins_g | 0 | 0 | 0 | 0 | 1 | ## |  | 0 | 0 | 0 | 0 | 0 | 1 |
| katG 371_del_g | 0 | 0 | 0 | 0 | 0 | 0 |  | 0 | 0 | 0 | 1 | 100 | 1 |
| katG 402_del_cgacaattcgc; katG 177_del_tgggtagcccagtc | 0 | 0 | 0 | 0 | 0 | 0 |  | 0 | 0 | 0 | 1 | 100 | 1 |
| katG 521_ins_t | 0 | 0 | 0 | 0 | 0 | 0 | 1 | 100 | 0 | 0 | 0 | 0 | 1 |
| katG 540_del_gcagaacttc | 0 | 0 | 0 | 0 | 0 | 0 | 1 | 100 | 0 | 0 | 0 | 0 | 1 |
| katG 89_ins_g | 0 | 0 | 0 | 0 | 0 | 0 |  | 0 | 0 | 0 | 1 | 100 | 1 |
| katG 978_del_g | 0 | 0 | 0 | 0 | 0 | 0 | 1 | 100 | 0 | 0 | 0 | 0 | 1 |
| katG D142G | 0 | 0 | 0 | 0 | 0 | 0 | 1 | 100 | 0 | 0 | 0 | 0 | 1 |
| katG E334_ | 0 | 0 | 0 | 0 | 0 | 0 |  | 0 | 0 | 0 | 1 | 100 | 1 |
| katG G14_ | 0 | 0 | 0 | 0 | 0 | 0 |  | 0 | 0 | 0 | 1 | 100 | 1 |
| katG G279D | 0 | 0 | 0 | 0 | 0 | 0 | 1 | 100 | 0 | 0 | 0 | 0 | 1 |
| katG N138S | 1 | 100 | 0 | 0 | 0 | 0 |  | 0 | 0 | 0 | 0 | 0 | 1 |
| katG Q500_ | 0 | 0 | 0 | 0 | 0 | 0 | 1 | 100 | 0 | 0 | 0 | 0 | 1 |
| katG S315I | 1 | 25 | 0 | 0 | 0 | 0 | 1 | 25 | 0 | 0 | 2 | 50 | 4 |
| katG S315N | 1 | 8.3 | 0 | 0 | 0 | 0 | 8 | 66.7 | 0 | 0 | 3 | 25 | 12 |
| **katG S315T** | 3 | 3 | 19 | 19 | 0 | 0 | 64 | 64.6 | 0 | 0 | 13 | 13 | 99 |
| **katG S315T**; katG 15_del_caca | 0 | 0 | 0 | 0 | 0 | 0 |  | 0 | 0 | 0 | 1 | 100 | 1 |
| katG V1A | 0 | 0 | 0 | 0 | 0 | 0 | 1 | 100 | 0 | 0 | 0 | 0 | 1 |
| katG W412C | 0 | 0 | 0 | 0 | 0 | 0 |  | 0 | 0 | 0 | 1 | 100 | 1 |
| katG W668_ | 0 | 0 | 0 | 0 | 0 | 0 | 1 | 100 | 0 | 0 | 0 | 0 | 1 |
| Grand Total | 10 |  | 22 |  | 2 |  | 147 |  | 7 |  | 50 |  | 238 |
