# Supplementary material for: Molecular determinants of multidrug-resistant tuberculosis in Sierra Leone
Source: Microbiol Spectr. 2024 Jan 30;12(3):e02405-23. doi: 10.1128/spectrum.02405-23 (PMC10923214; doi:10.1128/spectrum.02405-23)
Supplement: Figures S5, S6, S7, S8 — Supplementary figures. [file spectrum.02405-23-s0002.pdf]

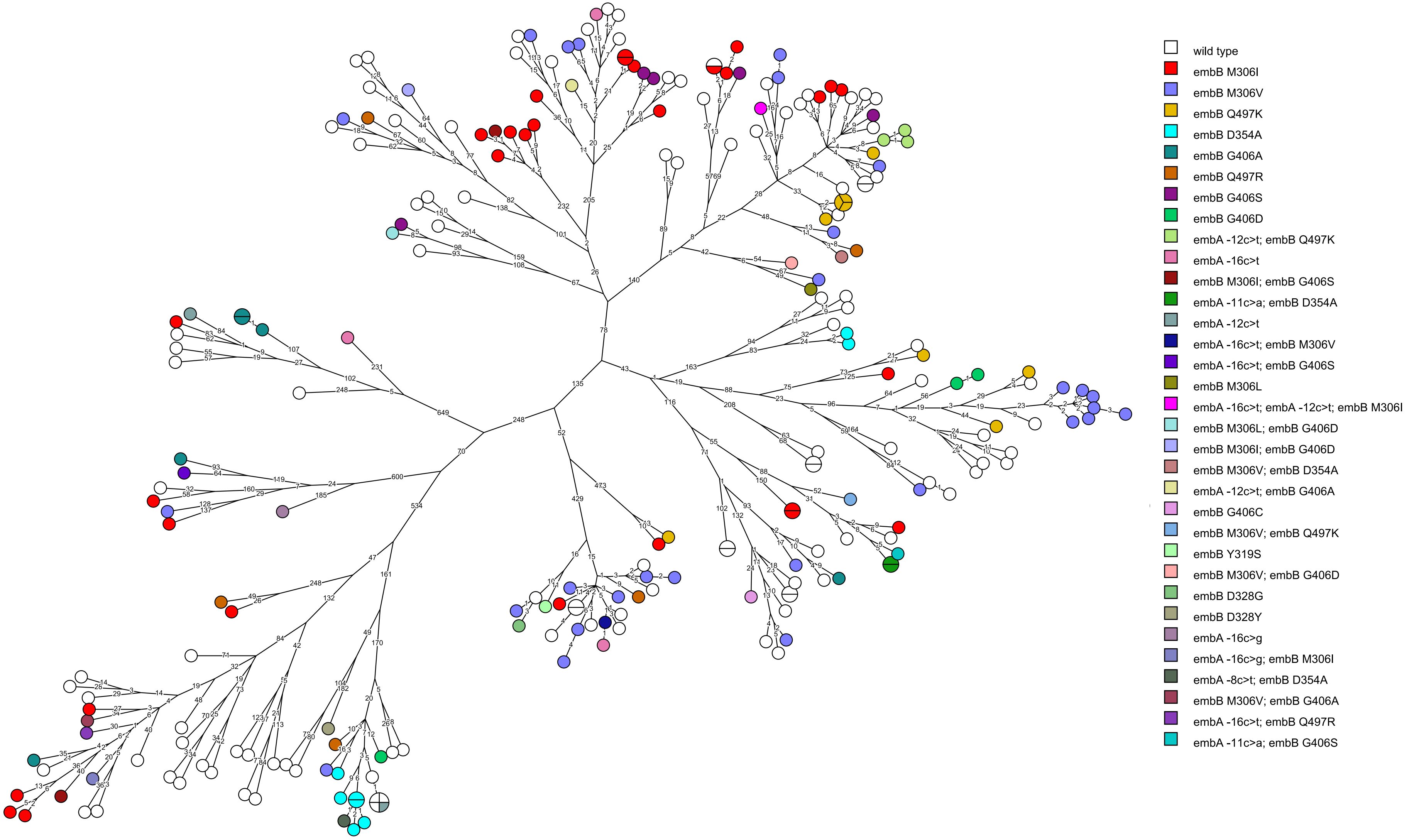

Figure S5. Maximum parsimony tree of 238 Sierra Leone MTBC strains displaying the ethambutol resistance mutations. The different mutations detected are color-coded.

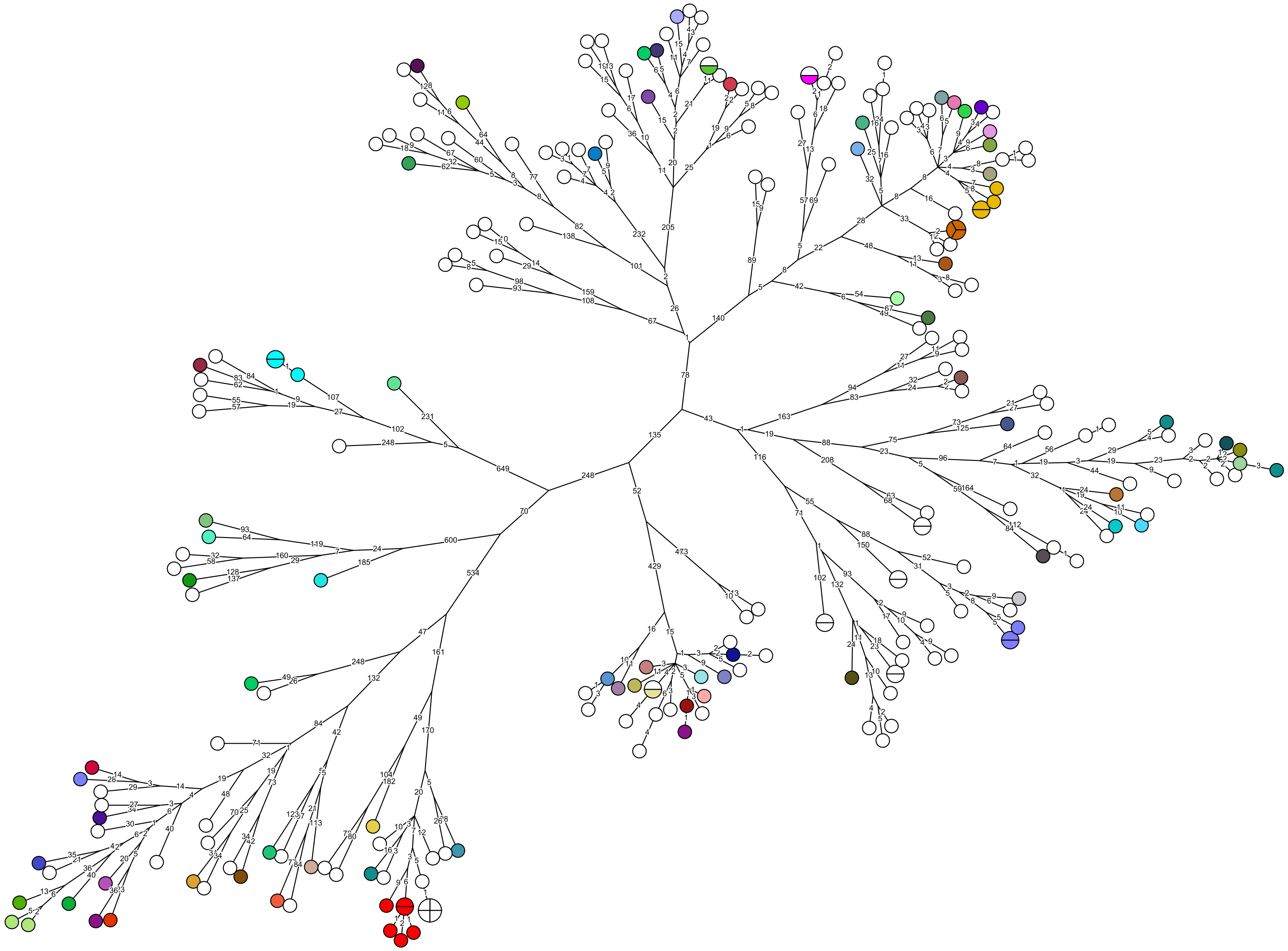

wild type
pncA D12A
pncA Q10P
pncA L172P
pncA S104R
pncA -11a>g
pncA V130A
pncA D136G
pncA H57P
pncA P62S
pncA V163G
pncA I133T; pncA H71P
pncA V9A
pncA T76I
pncA L182S; pncA V157G; pncA S104R; pncA V7F; pncA -11a>g
pncA K96T; pncA T76P
pncA L182W; pncA 197_del_ctccttatcaggccacacggcctcttcaccagtgggcccagctacac; pncA
pncA V7A; pncA -11a>g
pncA Q141_
pncA V7G
pncA H71Q; pncA P69S
pncA A146T; pncA W119C; pncA A102P; pncA T76P; pncA C72R
pncA G17D
pncA K96E
pncA F94L
pncA F58L; pncA L4W
pncA V180F
pncA G108R; pncA Y103_; pncA 48_del_tgggagc
pncA -7t>c
pncA T153N; pncA R148P; pncA G97C; pncA I6L
pncA G162A; pncA A146T; pncA D12A
pncA M175R; pncA L151S; pncA V130A; pncA 187_del_g; pncA H51Y
pncA S164P
pncA L172P; pncA 454_del_g; pncA V139A; pncA S67P; pncA L19P; pncA V7G; and oth
pncA 417_del_gtgt
pncA 395_ins_gctggtgta
pncA F81C
pncA D8Y
pncA H137N
pncA T100I; pncA -11a>g
pncA G105C
pncA A146P; pncA D136N; pncA D63Y
pncA T142M; pncA 335_del_aag; pncA V9A
pncA A92P
pncA T135P; pncA V125F; pncA S104I; pncA D12A; pncA V9A
pncA D49Y
pncA 464_del_tgggaccaccggtt; pncA D136A; pncA L85P; pncA -11a>c
pncA 523_ins_gag
pncA V180F; pncA Y103S; pncA K48E
pncA Y64S; pncA 86_ins_c
pncA W68R; pncA 173_del_tt; pncA D12A
pncA P69L
pncA E144_
pncA A146V
pncA S104I; pncA T47I
pncA R121P; pncA Y103H; pncA Y64_; pncA H51Q
pncA S59P
pncA R154W
pncA G97S; pncA -11a>g
pncA Y103C; pncA S59F
pncA P54L
pncA K48T; pncA -11a>c
pncA L172P; pncA 496_ins_c
pncA V128L
pncA 336_ins_a
pncA 191_del_atcaggcc; pncA D8N
pncA 452_del_t
pncA V139M; pncA A46V
pncA W119L
pncA C14R
pncA F81V; pncA -11a>g
pncA K96T; pncA -11a>g
pncA D12G

Figure S6. Maximum parsimony tree of 250 Sierra Leone MTBC strains displaying the pyrazinamide resistance mutations. The different mutations detected are color-coded.

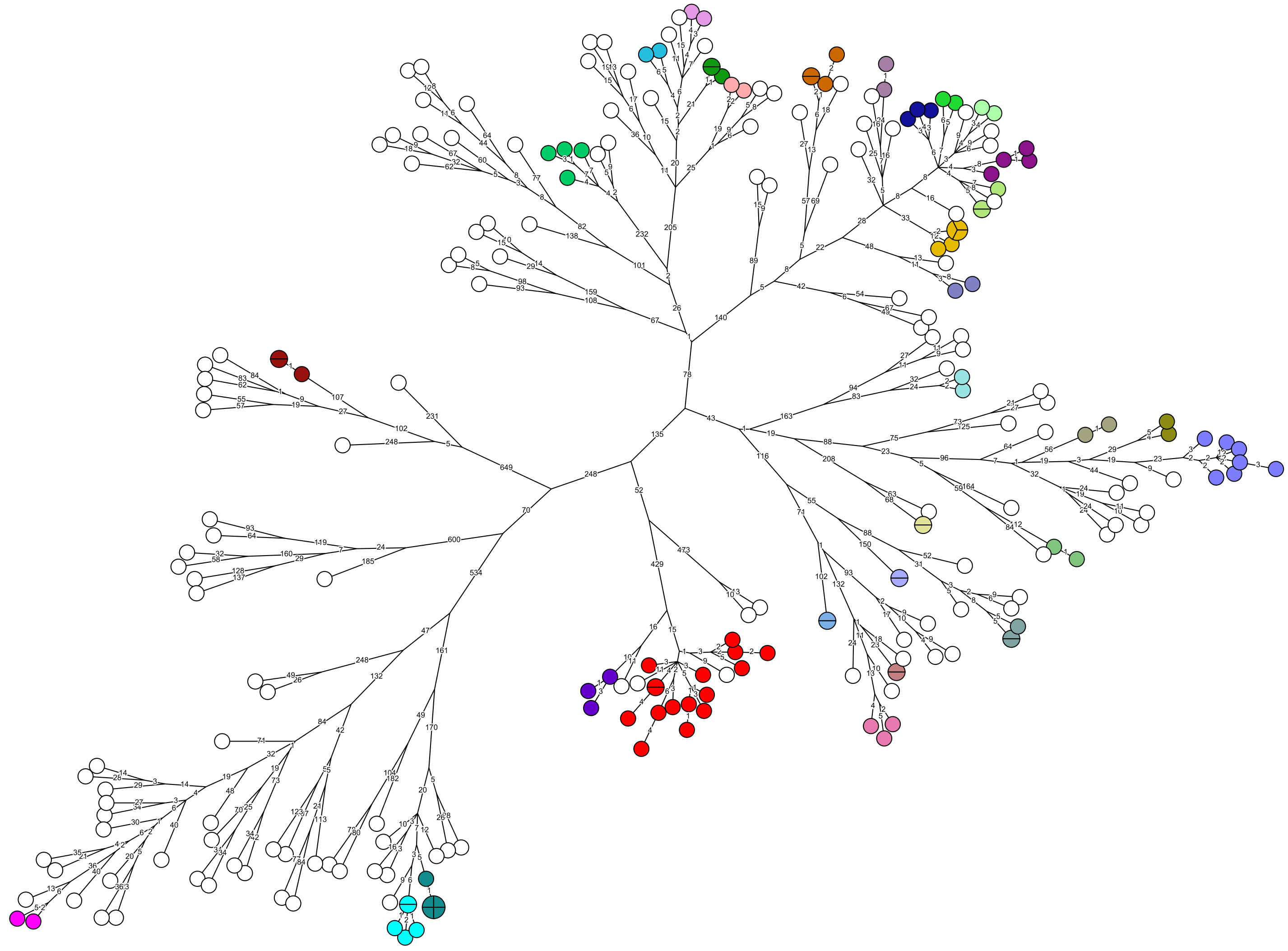

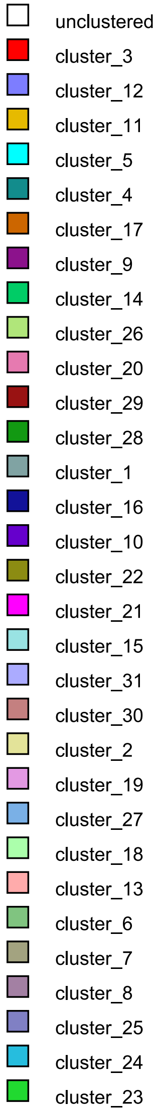

Figure S7. Maximum parsimony tree of 238 Sierra Leone strains grouped based on ≤12 SNP distance apart from each other. The different clusters are color-coded.

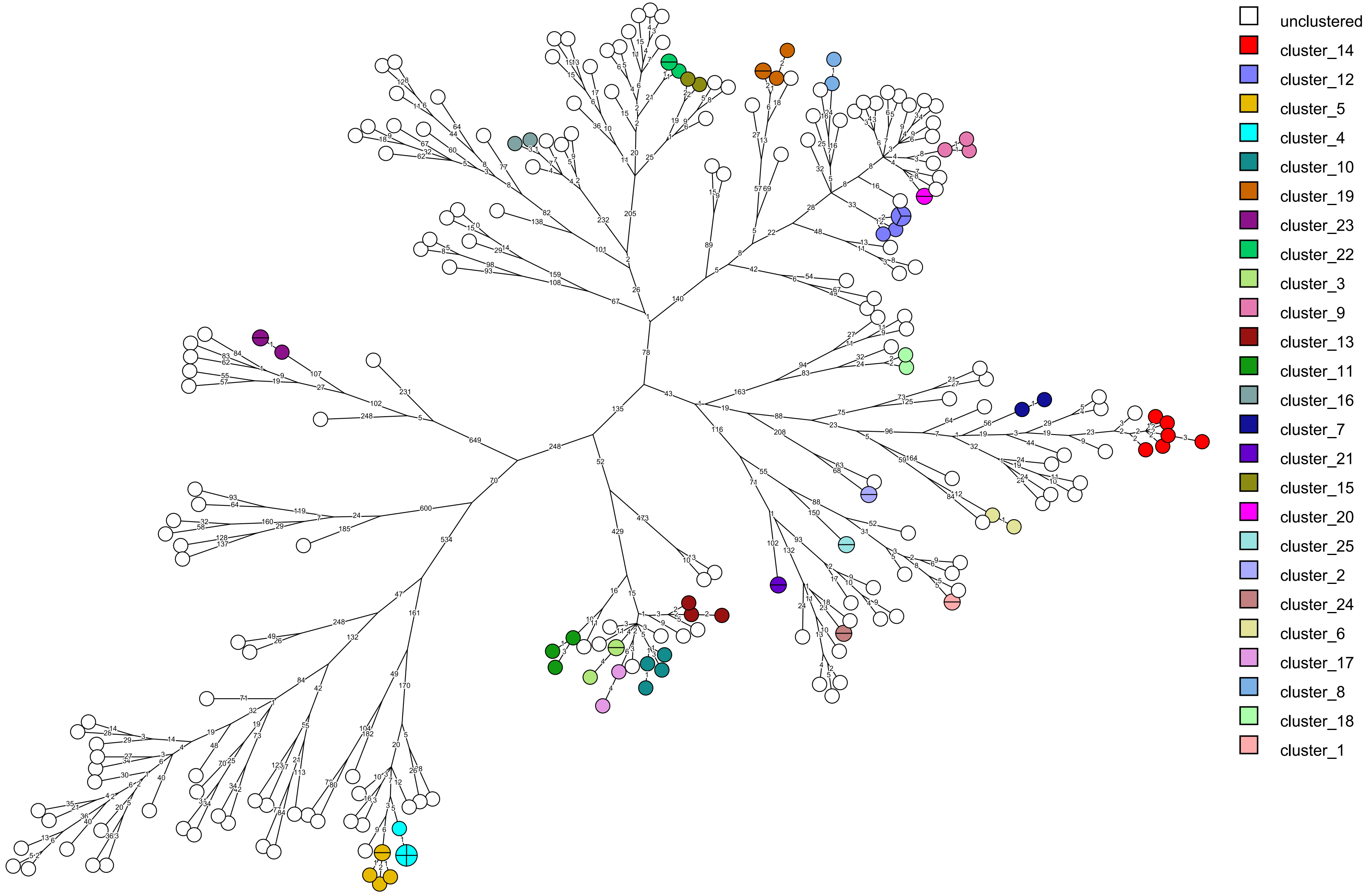

Figure S8. Maximum parsimony tree of 238 Sierra Leone strains grouped based on ≤5 distance apart from each other. The different clusters are color-coded
