# Supplementary material for: Molecular determinants of multidrug-resistant tuberculosis in Sierra Leone
Source: Microbiol Spectr. 2024 Jan 30;12(3):e02405-23. doi: 10.1128/spectrum.02405-23 (PMC10923214; doi:10.1128/spectrum.02405-23)
Supplement: Figures S1, S2, S3, S4 — Supplementary figures. [file spectrum.02405-23-s0003.pdf]

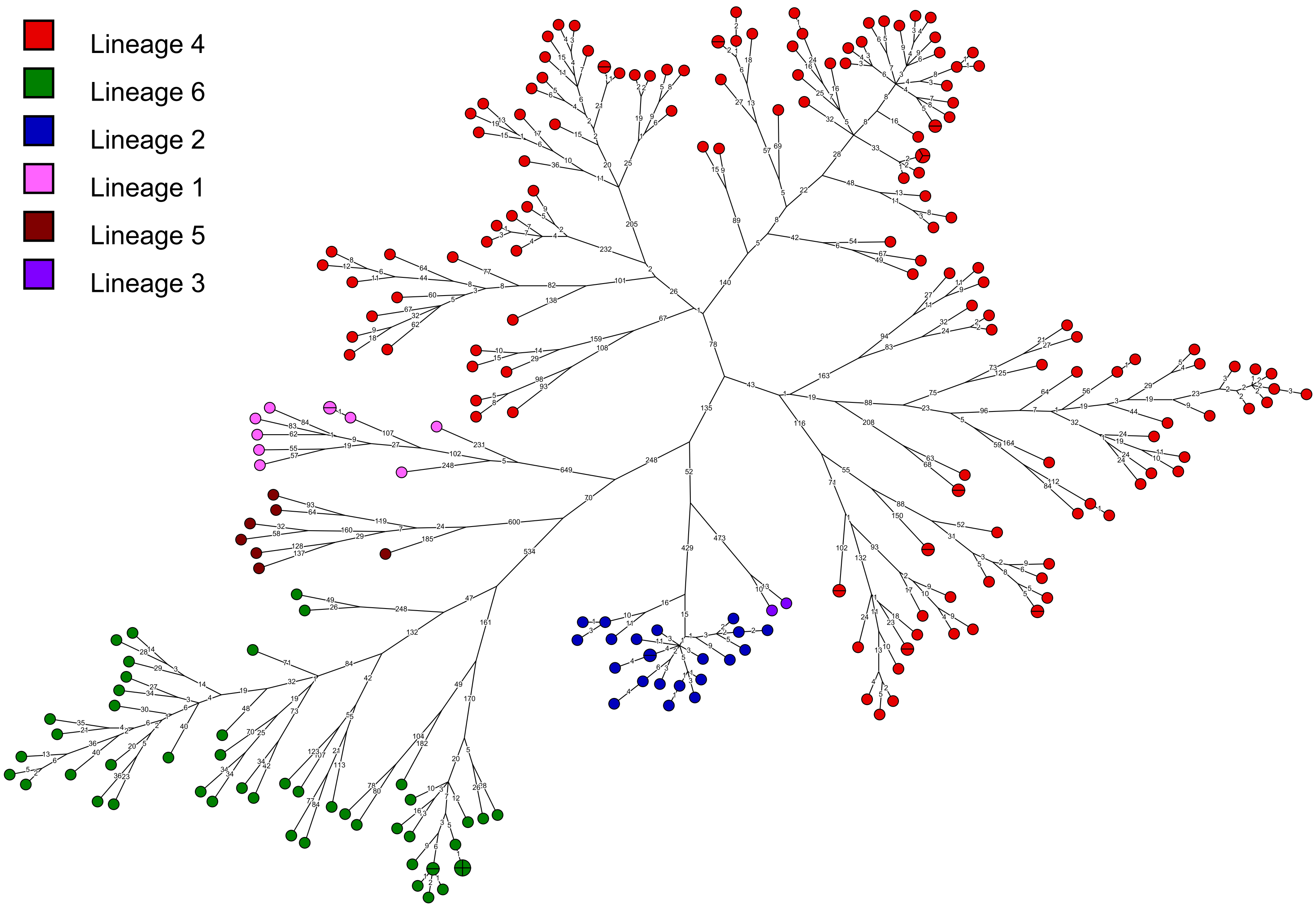

Figure S1. Lineage distribution of MTBC strains on maximum parsimony tree of 250 strains from Sierra Leone built from *16 936* SNP positions with Bionumerics 7.6. The different MTBC lineages are color-coded.

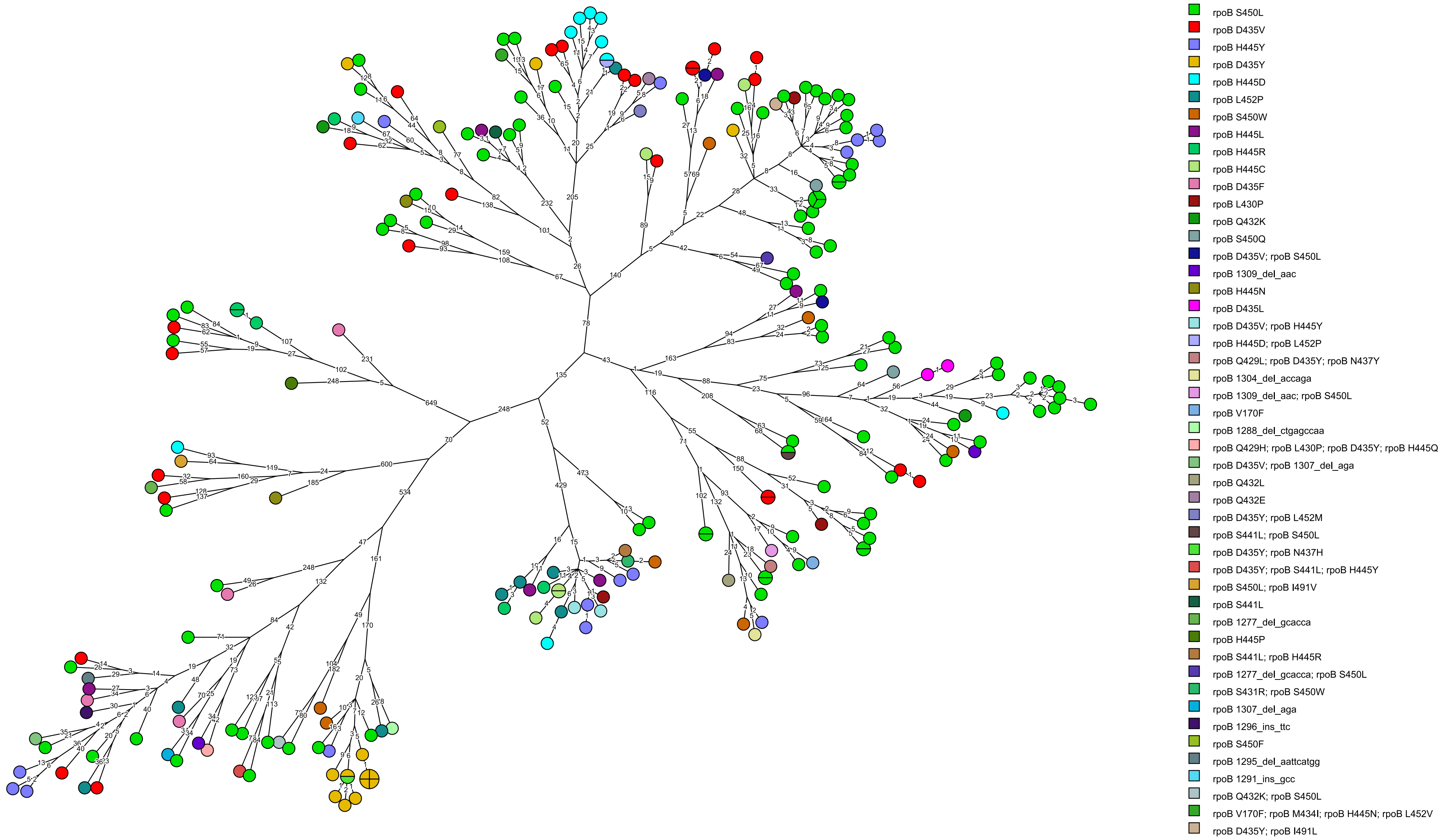

Figure S2. Maximum parsimony tree of 238 Sierra Leone MTBC strains displaying the rifampicin resistance mutations. The different mutations detected are color-coded.

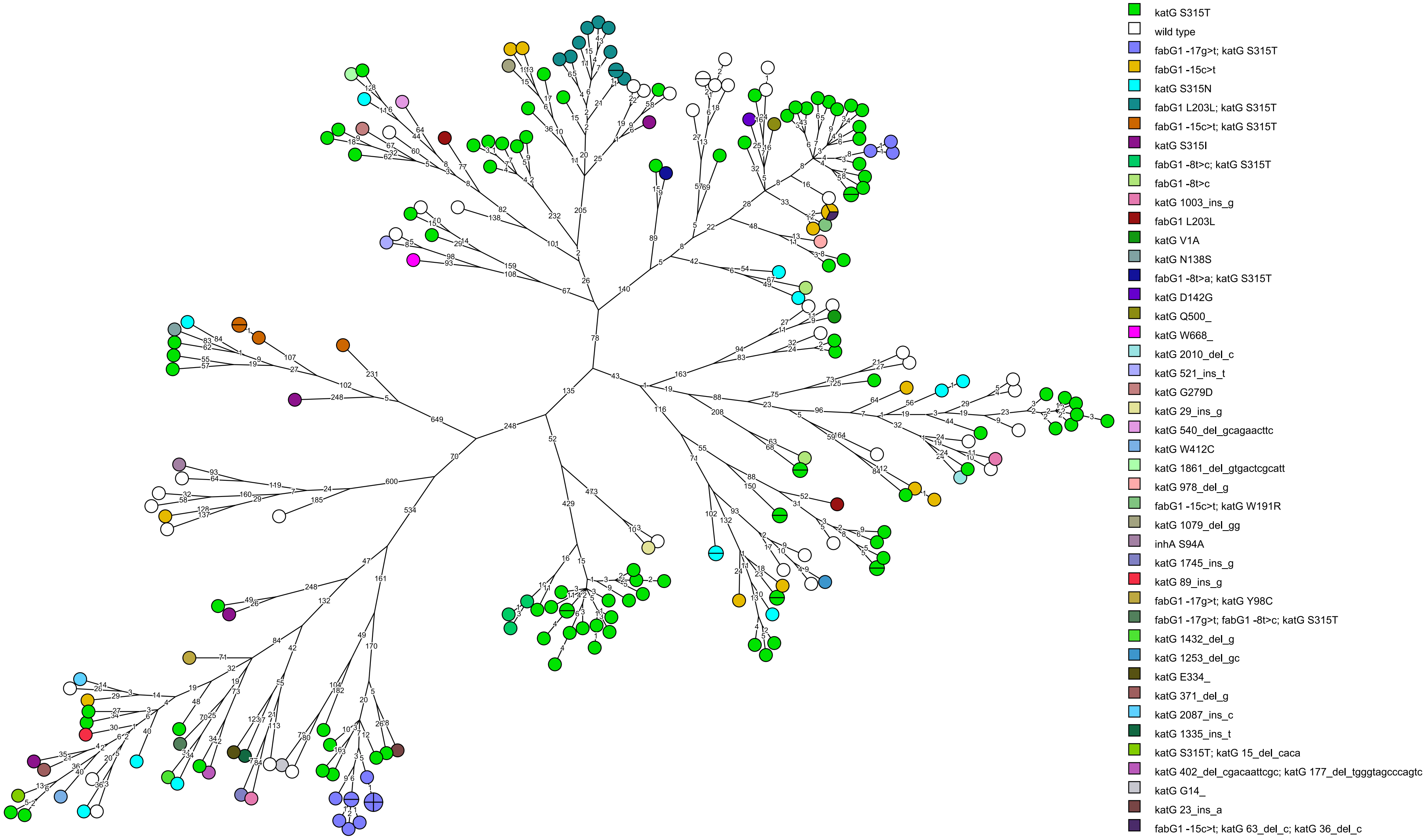

Figure S3. Maximum parsimony tree of 238 Sierra Leone MTBC strains displaying the isoniazid resistance mutations. The different mutations detected are color-coded.

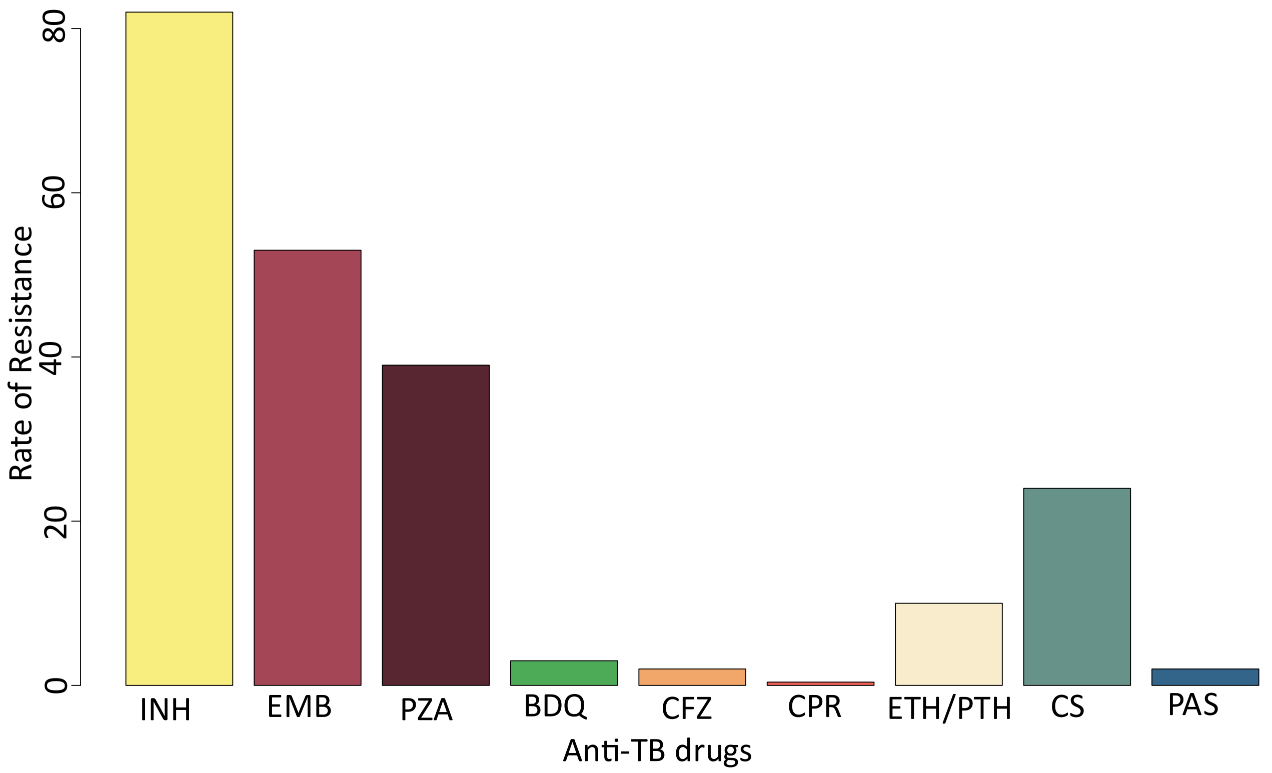

Figure S4. Proportion of isolates resistant to anti-TB drugs: Each drug is color-coded. RMP= rifampicin, INH= isoniazid, EMB= ethambutol, PZA= pyrazinamide, CS=cycloserine, ETH= ethionamide, PAS= para-aminosalicylic acid, CFZ=cloflaminine, BDQ= bedaquiline, KAN= kanamycin, CPR= capreomycin
