# Supplementary material for: Molecular determinants of multidrug-resistant tuberculosis in Sierra Leone
Source: Microbiol Spectr. 2024 Jan 30;12(3):e02405-23. doi: 10.1128/spectrum.02405-23 (PMC10923214; doi:10.1128/spectrum.02405-23)
Supplement: Table S2 — Supplementary table. [file spectrum.02405-23-s0005.docx]

Supplementary table S2.

Supplementary table S2.1. Sublineage distribution of MTBC strains from Sierra leone

| **Sublineage** | **Number** | **%** |
| --- | --- | --- |
| 5 West Africa 1 | 1 | 0.4 |
| 1.1.1 EAI | 10 | 4.2 |
| 2.2.1 Beijing Ancestral 3 | 22 | 9.2 |
| 3 Delhi-CAS | 2 | 0.8 |
| 4.1 Euro-American | 38 | 16.0 |
| 4.1.1.1 X-type | 3 | 1.3 |
| 4.1.1.3 X-type | 3 | 1.3 |
| 4.1.2.1 Haarlem | 45 | 18.9 |
| 4.3.3 LAM | 14 | 5.9 |
| 4.3.4.1 LAM | 2 | 0.8 |
| 4.3.4.2 LAM | 7 | 2.9 |
| 4.4.1.1 S-type | 6 | 2.5 |
| 4.6.2.2 Cameroon | 3 | 1.3 |
| 4.8 mainly T | 23 | 9.7 |
| 4.9 H37Rv-like | 3 | 1.3 |
| 5 West Africa 1 | 2 | 0.8 |
| 5.1.3 West Africa 1 | 1 | 0.4 |
| 5.1.5 West Africa 1 | 2 | 0.8 |
| 5.3 West Africa 1 | 1 | 0.4 |
| 6.1.3 West Africa 2 | 2 | 0.8 |
| 6.2.2 West Africa 2 | 23 | 9.7 |
| 6.2.3 West Africa 2 | 5 | 2.1 |
| 6.3.1 West Africa 2 | 3 | 1.3 |
| 6.3.3 West Africa 2 | 17 | 7.1 |
| Percentages are based on overall number of strains, 238 | | |

Supplementary table S2.2. Drug Resistance proportions

| **Drug** | **no. resistant** | **%** |
| --- | --- | --- |
| RR | 238 | 100 |
| INH | 196 | 82 |
| EMB | 126 | 53 |
| PZA | 92 | 39 |
| BDQ | 6 | 3 |
| CFZ | 5 | 2 |
| ETH/PTH | 23 | 10 |
| CPR | 1 | 0 |
| CS | 57 | 24 |
| PAS | 5 | 2 |
| % denominator = 238 | |  |

Supplementary table S2.3. Distribution of strains based on treatment

|  | **Number** | **%** |
| --- | --- | --- |
| MDR | 196 | 82.4 |
| 1st-line | 61 | 25.6 |
| MDR+BDQ +CFZ | 5 | 2.1 |
| 1st-line + BDQ + CFZ | 3 | 1.3 |
| Ist-line + BDQ + SM | 2 | 0.8 |
| % denominator = 238 | |  |

Supplementary table S2.4. Distribution based on lineage and resistances

|  | **n** | **L1 (%)** | **L2(%)** | **L3(%)** | **L4(%)** | **L5 (%)** | **L6 (%)** |
| --- | --- | --- | --- | --- | --- | --- | --- |
| MDR | 196 | 10(5.1) | 22(11.2) | 1(0.5) | 116(59.1) | 2(1) | 45(23) |
| 1st-line | 61 | 5(8.2) | 8(13.1) | 0 | 31(50.8) | 2(3.2) | 15(24.6) |
| BDQ+CFZ | 5 | 1(20) | 1(20) | 0 | 1(20) | 0 | 2(40) |
| MDR+BDQ +CFZ | 5 | 1(20) | 1(20) | 0 | 1(20) | 0 | 2(40) |
| 1st-line + BDQ + CFZ | 3 | 1(33.3) | 0.0 | 0 | 0 | 0 | 2(66.7) |
| Ist-line + BDQ + SM | 2 | 0 | 0.0 | 0 | 0 | 0 | 2(100) |
| Percentages are based on horizontal total | | |  |  |  |  |  |

Supplementary table S2.5. Cluster rate based on ≤12 SNP distances within the sublineages

| **Sublineage** | **# clustered** | **Total** | **%** |
| --- | --- | --- | --- |
| 5 West Africa 1 | 0 | 1 | 0.0 |
| 1.1.1 EAI | 3 | 10 | 30.0 |
| 2.2.1 Beijing Ancestral 3 | 19 | 22 | 86.4 |
| 3 Delhi-CAS |  | 2 | 0.0 |
| 4.1 Euro-American | 13 | 38 | 34.2 |
| 4.1.1.1 X-type |  | 3 | 0.0 |
| 4.1.1.3 X-type |  | 3 | 0.0 |
| 4.1.2.1 Haarlem | 28 | 45 | 62.2 |
| 4.3.3 LAM | 7 | 14 | 50.0 |
| 4.3.4.1 LAM | 2 | 2 | 100.0 |
| 4.3.4.2 LAM | 3 | 7 | 42.9 |
| 4.4.1.1 S-type | 2 | 6 | 33.3 |
| 4.6.2.2 Cameroon | 2 | 3 | 66.7 |
| 4.8 mainly T | 13 | 23 | 56.5 |
| 4.9 H37Rv-like |  | 3 | 0.0 |
| 5 West Africa 1 |  | 2 | 0.0 |
| 5.1.3 West Africa 1 |  | 1 | 0.0 |
| 5.1.5 West Africa 1 |  | 2 | 0.0 |
| 5.3 West Africa 1 |  | 1 | 0.0 |
| 6.1.3 West Africa 2 |  | 2 | 0.0 |
| 6.2.2 West Africa 2 | 2 | 23 | 8.7 |
| 6.2.3 West Africa 2 |  | 5 | 0.0 |
| 6.3.1 West Africa 2 |  | 3 | 0.0 |
| 6.3.3 West Africa 2 | 10 | 17 | 58.8 |
| Percentages are based on the total number of strains per sublineage | | |  |
