# Supplementary material for: Molecular determinants of multidrug-resistant tuberculosis in Sierra Leone
Source: Microbiol Spectr. 2024 Jan 30;12(3):e02405-23. doi: 10.1128/spectrum.02405-23 (PMC10923214; doi:10.1128/spectrum.02405-23)
Supplement: Table S3 — Supplementary table. [file spectrum.02405-23-s0006.docx]

Supplementary table 3

Supplementary table S3.1. RIF resistance mutations

| **mutations** | **No.** | **%** |
| --- | --- | --- |
| rpoB 1277_del_gcacca | 1 | 0.42 |
| rpoB 1277_del_gcacca; rpoB S450L | 1 | 0.42 |
| rpoB 1288_del_ctgagccaa | 1 | 0.42 |
| rpoB 1291_ins_gcc | 1 | 0.42 |
| rpoB 1295_del_aattcatgg | 1 | 0.42 |
| rpoB 1296_ins_ttc | 1 | 0.42 |
| rpoB 1304_del_accaga | 1 | 0.42 |
| rpoB 1307_del_aga | 1 | 0.42 |
| rpoB 1309_del_aac | 2 | 0.84 |
| rpoB 1309_del_aac; rpoB S450L | 1 | 0.42 |
| rpoB D435F | 4 | 1.68 |
| rpoB D435L | 2 | 0.84 |
| rpoB D435V | 25 | 10.50 |
| rpoB D435V; rpoB 1307_del_aga | 1 | 0.42 |
| rpoB D435V; rpoB H445Y | 2 | 0.84 |
| rpoB D435V; rpoB S450L | 2 | 0.84 |
| rpoB D435Y | 13 | 5.46 |
| rpoB D435Y; rpoB I491L | 1 | 0.42 |
| rpoB D435Y; rpoB L452M | 1 | 0.42 |
| rpoB D435Y; rpoB N437H | 1 | 0.42 |
| rpoB D435Y; rpoB S441L; rpoB H445Y | 1 | 0.42 |
| rpoB H445C | 5 | 2.10 |
| rpoB H445D | 9 | 3.78 |
| rpoB H445D; rpoB L452P | 1 | 0.42 |
| rpoB H445L | 6 | 2.52 |
| rpoB H445N | 2 | 0.84 |
| rpoB H445P | 1 | 0.42 |
| rpoB H445R | 6 | 2.52 |
| rpoB H445Y | 15 | 6.30 |
| rpoB L430P | 3 | 1.26 |
| rpoB L452P | 8 | 3.36 |
| rpoB Q429H; rpoB L430P; rpoB D435Y; rpoB H445Q | 1 | 0.42 |
| rpoB Q429L; rpoB D435Y; rpoB N437Y | 1 | 0.42 |
| rpoB Q432E | 1 | 0.42 |
| rpoB Q432K | 2 | 0.84 |
| rpoB Q432K; rpoB S450L | 1 | 0.42 |
| rpoB Q432L | 1 | 0.42 |
| rpoB S431R; rpoB S450W | 1 | 0.42 |
| rpoB S441L | 1 | 0.42 |
| rpoB S441L; rpoB H445R | 1 | 0.42 |
| rpoB S441L; rpoB S450L | 1 | 0.42 |
| rpoB S450F | 1 | 0.42 |
| rpoB S450L | 94 | 39.50 |
| rpoB S450L; rpoB I491V | 1 | 0.42 |
| rpoB S450Q | 2 | 0.84 |
| rpoB S450W | 7 | 2.94 |
| rpoB V170F | 1 | 0.42 |
| rpoB V170F; rpoB M434I; rpoB H445N; rpoB L452V | 1 | 0.42 |
| **Total** | **238** |  |
| RIF = rifampicin |  |  |

Supplementary table S3.2. INH resistance mutations

| **mutations** | **No.** | **%** |
| --- | --- | --- |
| fabG1 -15c>t | 12 | 6.12 |
| fabG1 -15c>t; katG 63_del_c; katG 36_del_c | 1 | 0.51 |
| fabG1 -15c>t; katG S315T | 4 | 2.04 |
| fabG1 -15c>t; katG W191R | 1 | 0.51 |
| fabG1 -17g>t; fabG1 -8t>c; katG S315T | 1 | 0.51 |
| fabG1 -17g>t; katG S315T | 14 | 7.14 |
| fabG1 -17g>t; katG Y98C | 1 | 0.51 |
| fabG1 -8t>a; katG S315T | 1 | 0.51 |
| fabG1 -8t>c | 2 | 1.02 |
| fabG1 -8t>c; katG S315T | 3 | 1.53 |
| fabG1 L203L | 2 | 1.02 |
| fabG1 L203L; katG S315T | 10 | 5.10 |
| inhA S94A | 1 | 0.51 |
| katG 1003_ins_g | 2 | 1.02 |
| katG 1079_del_gg | 1 | 0.51 |
| katG 1253_del_gc | 1 | 0.51 |
| katG 1335_ins_t | 1 | 0.51 |
| katG 1432_del_g | 1 | 0.51 |
| katG 1745_ins_g | 1 | 0.51 |
| katG 1861_del_gtgactcgcatt | 1 | 0.51 |
| katG 2010_del_c | 1 | 0.51 |
| katG 2087_ins_c | 1 | 0.51 |
| katG 23_ins_a | 1 | 0.51 |
| katG 29_ins_g | 1 | 0.51 |
| katG 371_del_g | 1 | 0.51 |
| katG 402_del_cgacaattcgc; katG 177_del_tgggtagcccagtc | 1 | 0.51 |
| katG 521_ins_t | 1 | 0.51 |
| katG 540_del_gcagaacttc | 1 | 0.51 |
| katG 89_ins_g | 1 | 0.51 |
| katG 978_del_g | 1 | 0.51 |
| katG D142G | 1 | 0.51 |
| katG E334_ | 1 | 0.51 |
| katG G14_ | 1 | 0.51 |
| katG G279D | 1 | 0.51 |
| katG N138S | 1 | 0.51 |
| katG Q500_ | 1 | 0.51 |
| katG S315I | 4 | 2.04 |
| katG S315N | 12 | 6.12 |
| **katG S315T** | 99 | 50.51 |
| **katG S315T**; katG 15_del_caca | 1 | 0.51 |
| katG V1A | 1 | 0.51 |
| katG W412C | 1 | 0.51 |
| katG W668_ | 1 | 0.51 |
| **Total** | **196** |  |
| INH = isoniazid |  |  |

Supplementary table S3.3 EMB resistance mutations

| **mutations** | **No.** | **%** |
| --- | --- | --- |
| embA -11c>a; embB D354A | 2 | 1.59 |
| embA -11c>a; embB G406S | 1 | 0.79 |
| embA -12c>t | 2 | 1.59 |
| embA -12c>t; embB G406A | 1 | 0.79 |
| embA -12c>t; embB Q497K | 3 | 2.38 |
| embA -16c>g | 1 | 0.79 |
| embA -16c>g; embB M306I | 1 | 0.79 |
| embA -16c>t | 3 | 2.38 |
| embA -16c>t; embA -12c>t; embB M306I | 1 | 0.79 |
| embA -16c>t; embB G406S | 1 | 0.79 |
| embA -16c>t; embB M306V | 1 | 0.79 |
| embA -16c>t; embB Q497R | 1 | 0.79 |
| embA -8c>t; embB D354A | 1 | 0.79 |
| embB D328G | 1 | 0.79 |
| embB D328Y | 1 | 0.79 |
| embB D354A | 8 | 6.35 |
| embB G406A | 6 | 4.76 |
| embB G406C | 1 | 0.79 |
| embB G406D | 3 | 2.38 |
| embB G406S | 5 | 3.97 |
| embB M306I | 30 | 23.81 |
| embB M306I; embB G406D | 1 | 0.79 |
| embB M306I; embB G406S | 2 | 1.59 |
| embB M306L | 1 | 0.79 |
| embB M306L; embB G406D | 1 | 0.79 |
| embB M306V | 28 | 22.22 |
| embB M306V; embB D354A | 1 | 0.79 |
| embB M306V; embB G406A | 1 | 0.79 |
| embB M306V; embB G406D | 1 | 0.79 |
| embB M306V; embB Q497K | 1 | 0.79 |
| embB Q497K | 9 | 7.14 |
| embB Q497R | 5 | 3.97 |
| embB Y319S | 1 | 0.79 |
| **Total** | **126** |  |
| EMB = ethambutol | |  |

Supplementary table S3.4 PZA resistance mutations

| **mutations** | **No.** | **%** |
| --- | --- | --- |
| pncA -11a>g | 3 | 3.26 |
| pncA 191_del_atcaggcc; pncA D8N | 1 | 1.09 |
| pncA 336_ins_a | 1 | 1.09 |
| pncA 395_ins_gctggtgta | 1 | 1.09 |
| pncA 417_del_gtgt | 1 | 1.09 |
| pncA 452_del_t | 1 | 1.09 |
| pncA 464_del_tgggaccaccggtt; pncA D136A; pncA L85P; pncA -11a>c | 1 | 1.09 |
| pncA 523_ins_gag | 1 | 1.09 |
| pncA -7t>c | 1 | 1.09 |
| pncA A146P; pncA D136N; pncA D63Y | 1 | 1.09 |
| pncA A146T; pncA W119C; pncA A102P; pncA T76P; pncA C72R | 1 | 1.09 |
| pncA A146V | 1 | 1.09 |
| pncA A92P | 1 | 1.09 |
| pncA C14R | 1 | 1.09 |
| pncA D12A | 6 | 6.52 |
| pncA D12G | 1 | 1.09 |
| pncA D136G | 2 | 2.17 |
| pncA D49Y | 1 | 1.09 |
| pncA D8Y | 1 | 1.09 |
| pncA E144_ | 1 | 1.09 |
| pncA F58L; pncA L4W | 1 | 1.09 |
| pncA F81C | 1 | 1.09 |
| pncA F81V; pncA -11a>g | 1 | 1.09 |
| pncA F94L | 1 | 1.09 |
| pncA G105C | 1 | 1.09 |
| pncA G108R; pncA Y103_; pncA 48_del_tgggagc | 1 | 1.09 |
| pncA G162A; pncA A146T; pncA D12A | 1 | 1.09 |
| pncA G17D | 1 | 1.09 |
| pncA G97S; pncA -11a>g | 1 | 1.09 |
| pncA H137N | 1 | 1.09 |
| pncA H57P | 2 | 2.17 |
| pncA H71Q; pncA P69S | 1 | 1.09 |
| pncA I133T; pncA H71P | 1 | 1.09 |
| pncA K48T; pncA -11a>c | 1 | 1.09 |
| pncA K96E | 1 | 1.09 |
| pncA K96T; pncA -11a>g | 1 | 1.09 |
| pncA K96T; pncA T76P | 1 | 1.09 |
| pncA L172P | 4 | 4.35 |
| pncA L172P; pncA 454_del_g; pncA V139A; pncA S67P; pncA L19P; pncA V7G; and others | 1 | 1.09 |
| pncA L172P; pncA 496_ins_c | 1 | 1.09 |
| pncA L182S; pncA V157G; pncA S104R; pncA V7F; pncA -11a>g | 1 | 1.09 |
| pncA L182W; pncA 197_del_ctccttatcaggccacacggcctcttcaccagtgggcccagctacac; pncA D49E | 1 | 1.09 |
| pncA M175R; pncA L151S; pncA V130A; pncA 187_del_g; pncA H51Y | 1 | 1.09 |
| pncA P54L | 1 | 1.09 |
| pncA P62S | 2 | 2.17 |
| pncA P69L | 1 | 1.09 |
| pncA Q10P | 4 | 4.35 |
| pncA Q141_ | 1 | 1.09 |
| pncA R121P; pncA Y103H; pncA Y64_; pncA H51Q | 1 | 1.09 |
| pncA R154W | 1 | 1.09 |
| pncA S104I; pncA T47I | 1 | 1.09 |
| pncA S104R | 3 | 3.26 |
| pncA S164P | 1 | 1.09 |
| pncA S59P | 1 | 1.09 |
| pncA T100I; pncA -11a>g | 1 | 1.09 |
| pncA T135P; pncA V125F; pncA S104I; pncA D12A; pncA V9A | 1 | 1.09 |
| pncA T142M; pncA 335_del_aag; pncA V9A | 1 | 1.09 |
| pncA T153N; pncA R148P; pncA G97C; pncA I6L | 1 | 1.09 |
| pncA T76I | 1 | 1.09 |
| pncA V128L | 1 | 1.09 |
| pncA V130A | 3 | 3.26 |
| pncA V139M; pncA A46V | 1 | 1.09 |
| pncA V163G | 1 | 1.09 |
| pncA V180F | 1 | 1.09 |
| pncA V180F; pncA Y103S; pncA K48E | 1 | 1.09 |
| pncA V7A; pncA -11a>g | 1 | 1.09 |
| pncA V7G | 1 | 1.09 |
| pncA V9A | 1 | 1.09 |
| pncA W119L | 1 | 1.09 |
| pncA W68R; pncA 173_del_tt; pncA D12A | 1 | 1.09 |
| pncA Y103C; pncA S59F | 1 | 1.09 |
| pncA Y64S; pncA 86_ins_c | 1 | 1.09 |
| **Total** | **92** |  |
| PZA = pyrazinamid | |  |

Supplementary table S3.5 BDQ resistance mutations

| **mutations** | **No.** | **%** |
| --- | --- | --- |
| Rv0678 138_ins_g; Rv0678 141_ins_c | 1 | 16.67 |
| Rv0678 193_del_g | 2 | 33.33 |
| Rv0678 95_ins_ga; Rv0678 Y92_ | 1 | 16.67 |
| Rv0678 C46R | 1 | 16.67 |
| Rv0678 R90C | 1 | 16.67 |
| **Total** | **6** |  |
| BDQ = bedaquiline | |  |

Supplementary table S3.6 CFZ resistance mutations

| **mutations** | **No.** | **%** |
| --- | --- | --- |
| Rv0678 138_ins_g; Rv0678 141_ins_c | 1 | 20 |
| Rv0678 193_del_g | 2 | 40 |
| Rv0678 95_ins_ga; Rv0678 Y92_ | 1 | 20 |
| Rv0678 R90C | 1 | 20 |
| **Total** | **5** |  |
| CFZ =clofazimine | |  |

Supplementary table S3.7 CS resistance mutations

| **mutations** | **No.** | **%** |
| --- | --- | --- |
| ald 266_del_a | 33 | 57.89 |
| ald 266_del_a; ald 366_del_cgccga | 7 | 12.28 |
| ald 266_del_a; ald 716_del_tcc | 17 | 29.82 |
| **Total** | **57** |  |
| CS = cycloserine | |  |
